# A Versatile High‐Throughput Single‐Cell Screening Platform for Profiling Antigen‐Specific Long‐Lived B Cells in Blood and Bone Marrow

**DOI:** 10.1002/advs.202414945

**Published:** 2025-04-09

**Authors:** Tian Zhao, Yuqing Lei, Chang Liu, Dong Zhang, Kaiyi Li, Sisi Shan, Chenyu Li, Zimeng Wei, Yuhan Yang, Ting Zhang, Kai Sun, Haoran Sun, Linqi Zhang, Peng Liu

**Affiliations:** ^1^ School of Biomedical Engineering Tsinghua University Beijing 100084 China; ^2^ Comprehensive AIDS Research Center Center for Global Health and Infectious Diseases Research NexVac Research Center Center for Infectious Diseases Research School of Basic Medical Sciences Tsinghua Medicine Tsinghua University Beijing 100084 China; ^3^ Tsinghua‐Peking Joint Center for Life Sciences Beijing 100084 China; ^4^ Institute of Biopharmaceutical and Health Engineering Tsinghua Shenzhen International Graduate School Tsinghua University Shenzhen 518055 China; ^5^ Institute of Biomedical Health Technology and Engineering Shenzhen Bay Laboratory Shenzhen 518132 China; ^6^ Changping Laboratory Beijing 102206 China

**Keywords:** antibody, B cells, high‐throughput, microarray, single‐cell sequencing

## Abstract

Antigen‐specific B cells play a crucial role in the long‐term immune response following infection or vaccination, differentiating into antibody‐secreting cells (ASCs) and memory B cells (MBCs). However, profiling ASCs is challenging primarily due to their lack of membrane‐bound surface B cell receptors. In this study, the Modular Superhydrophobic Microwell Array Chip (MoSMAR‐chip) is introduced as a versatile, cost‐effective, and high‐throughput platform for identifying and characterizing individual antigen‐specific ASCs and MBCs at the single‐cell level within seven days. Using this platform, comprehensive analyses of single ASCs could be performed from bone marrows of coronavirus disease 2019 (COVID‐19) vaccine‐immunized mice and a diverse set of antibodies capable of neutralizing the highly divergent JN1 variant of severe acute respiratory syndrome coronavirus 2 (SARS‐CoV‐2) were identified. These results demonstrate that the MoSMAR‐chip facilitates efficient single‐cell multi‐omics and functional analyses of antigen‐specific ASCs, offering a powerful tool for investigating complex long‐term B cell immunity in diverse clinical conditions, such as infectious diseases, autoimmunity, and beyond.

## Graphical abstract

The MoSMAR‐chip platform provides a high‐throughput and cost‐effective tool for identifying and characterizing antigen‐specific antibody‐secreting cells (ASCs) and memory B cells (MBCs) at the single‐cell level. The ASCs from coronavirus disease 2019 (COVID‐19) vaccine‐immunized mice were successfully analyzed, revealing diverse neutralizing antibodies against the JN1 variant. The platform enables efficient single‐cell multi‐omics and functional studies, advancing B cell immunity research.

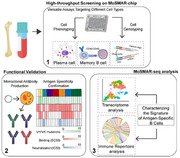

## 1 Introduction

The long‐term protective effects of vaccines are critically dependent on the sustained response of memory B cells (MBCs) and long‐lived plasma cells (LLPCs).^[^ ^1^ ^]^ MBCs contain B cell receptors (BCR) on their membrane surface and can rapidly differentiate into antibody‐secreting cells (ASC) upon re‐exposure to an antigen,^[^ ^2^ ^]^ while LLPCs reside in the bone marrow and continuously secrete antibodies to maintain the active humoral immune response.^[^ ^3^ ^]^ The ability to isolate and profile these antigen‐specific B cells at a single‐cell level is essential for understanding their roles in long‐term immunity and for developing effective vaccines and antibody‐based therapies. Current high‐throughput technologies have been successful in analyzing MBCs, leading to significant advances in understanding the immune response to vaccines and infections. These technologies, which include flow cytometry and single‐cell RNA sequencing, have enabled the identification of potent neutralizing antibodies and have played a key role in therapeutic antibody discovery. However, the profiling of LLPCs remains a challenge, largely due to the lack of membrane‐bound surface B cell receptors, making it impossible to directly labeling and identifying these cells.^[^ ^4^ ^]^

Recent attempts to overcome these limitations have involved the development of methods that encapsulate single cells in microdroplets or spatially confine secreted antibodies for detection.^[^ ^5^ ^]^ While these approaches have improved the ability to screen for antigen‐specific ASCs, they often require complex equipment, suffer from low cell capture rates, and pose difficulties in recovering cells for downstream analyses. Furthermore, many single‐cell sequencing methods are generally not well‐suited for integrating phenotypic screening with comprehensive genomic and transcriptomic profiling at the single‐cell level. For instance, addressable sequencing methods, while able to link phenotype to its genotype at single cell level, approve difficult for functional screening assays.^[^ ^6^ ^]^ Furthermore, plate‐based sequencing methods are often associated with high cost and complex manual operation steps that restrain their scale‐up and wide applications.^[^ ^7^ ^]^

In this study, we introduce the Modular Superhydrophobic Microwell Array Chip (MoSMAR‐chip), a versatile and high‐throughput platform designed to overcome the limitations of existing single‐cell screening technologies. The MoSMAR‐chip enables the identification, isolation, and sequencing of individual antigen‐specific B cells, including both MBCs and LLPCs, within a seven‐day timeframe. This platform allows for comprehensive antibody repertoire and transcriptome analyses, providing a powerful tool for understanding the role of LLPCs in long‐term immunity and for accelerating the discovery of therapeutic antibodies. We applied the MoSMAR‐chip platform to study the immune responses of mice immunized with a coronavirus disease 2019 (COVID‐19) vaccine, focusing on the identification of LLPCs that produce antibodies capable of neutralizing various severe acute respiratory syndrome coronavirus 2 (SARS‐CoV‐2) variants, including the JN1 strain. Our findings demonstrate that MoSMAR‐chip platform is a potent tool for linking phenotypic screening with genotypic analysis at the single‐cell level, offering new insights into the dynamics of long‐term B cell immunity and contributing to the development of effective vaccines and antibody therapies.

## 2 Results

### 2.1 Concept of MoSMAR System for Single‐Cell Antibody Capture and Screening

**Figure 1:**
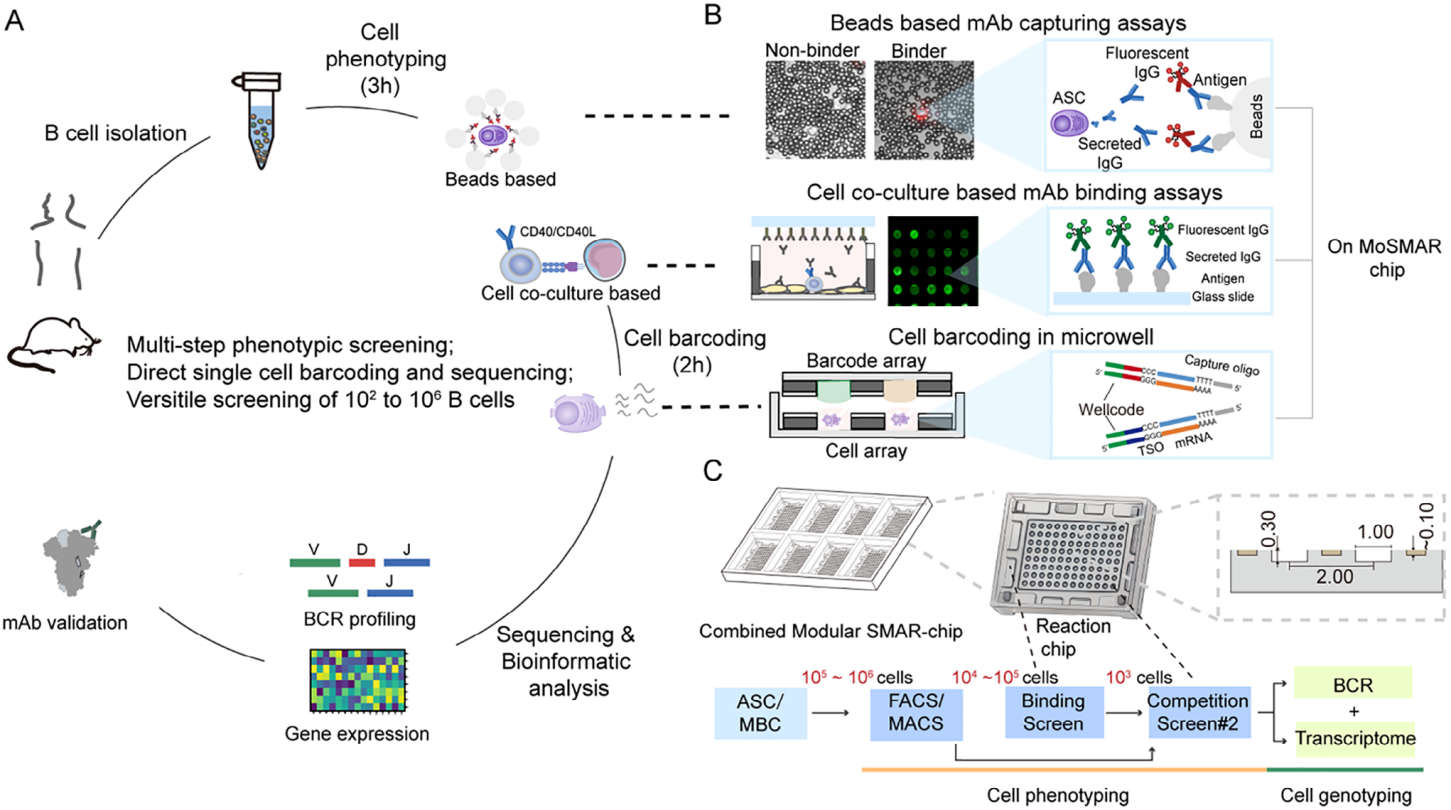
The overall workflow of MoSMAR‐chip system for isolation and characterization of mAbs from ASCs and memory B cells. A) Rapid discovery of mAb from ASCs and memory B cells using MoSMAR‐chip system. Mice or human B cells enriched from the peripheral blood mononuclear cells (PBMCs) or bone marrow mononuclear cells (BMMCs) were loaded onto the MoSMAR‐chip for phenotypic and genotypic screening. B cells producing desired antibodies were identified and sequenced, the corresponding B cell receptors (BCRs) were cloned for further validation. B) Underlying rational in beads‐ or co‐culture‐based assays for screening ASCs (upper panel) or memory B cells (lower panel), followed by capturing of mRNA and barcoding (bottom panel) of the selected ASCs and memory B cells. C) MoSMAR‐chip enables phenotyping and genotyping of ASCs and memory B cells with high versatility. Screening process initiates with ≈10^5^–10^6^ ASCs or memory B cells, followed by phenotyping of secreted antibodies, and finally genotyping of BCR and transcriptome of corresponding antigen‐specific ASCs or memory B cells. The whole procedure was performed on the MoSMAR‐chip.

To achieve the rapid screening and the analysis of B cells secreting antigen‐specific antibodies, we employed our previously developed modular superhydrophobic microwell array chip (MoSMAR‐chip) and single‐cell distribution platform (SCDI^[^ ^8^ ^]^) (**Figure** **1**; Figure S1, Supporting Information). The MoSMAR‐chip is a modular superhydrophobic microwell array designed for high‐throughput single‐cell screening, and the SCDI enables rapid, automated isolation of individual cells into microwells, significantly improving the throughput and accuracy. As shown in Figure S1A–C (Supporting Information), a single chip comprises 8 transfer wells surrounding an array of 96 microwells (arranged in 12 rows by 8 columns) segmented by superhydrophobic materials, assuring independent liquid condition in each microwell. B cells derived from mouse or human bone marrow or blood samples, including memory B cells (MBCs) and antibody secreting cells (ASCs), were loaded onto the chip and underwent multiple rounds of functional screening. Target B cells producing antibodies against specific antigens were picked and isolated into microwells for on‐chip single‐cell 5′‐RNA sequencing (Figure 1A). By recognizing the distinct characteristics of MBCs and ASCs, we designed different screening strategies to allow versatile B cell selection (Figure 1B). Specifically, for antigen‐specific ASCs, we use antigen‐coated beads surrounding the ASC as indicators to evaluate their antigen‐specificity. If secreted antibodies bind to the surrounding antigens on the beads, the ASCs could then be detected using fluorescent secondary antibodies, producing a halo‐like fluorescence pattern. For MBCs with membrane‐bound antibodies, we activate the cells and stimulate the antibody secretion through the CD40‐CD40L axis, and then analyzed the specificity and quantity of the secreted antibodies using antigen‐coated glass slide positioned on the top of the microwells. Both methods rely on antigen‐specific binding, ensuring accurate identification of individual B cells with appropriate antigen‐specificity.

Up to 16000 cells can be screened in a single transfer well, after which the cells of interest can be transferred to the microwells by the SCDI for further rounds of genetic and phenotypic characterizations. For samples with lower starting cell counts or those pre‐screened using fluorescence‐activated cell sorting (FACS) or magnetic‐activated cell sorting (MACS), individual cells can be directly distributed into the 96 microwell array (i.e., one cell per microwell). Thus, samples ranging from 10^2^ to 10⁶ cells can be processed on one set of MoSMAR‐chip (i.e., 8 single chips in the frame as shown in Figure S1B (Supporting Information) directly to isolate target cells for subsequent single‐cell multi‐omics analyses, including transcriptome and B cell receptor (BCR) profiling (Figure 1C). The superhydrophobic materials guarantee the independence of the microwells, and the use of mineral oil to seal the droplets further to ensure the stability of the reverse transcription reaction system (Figure S1D, Supporting Information). The MoSMAR‐chip platform also features a connection system via a 3D‐printed adapter that links the chip to conventional centrifuge tubes, allowing for the recovery of all droplets within 1 min through centrifugation (Figure S1E, Supporting Information). This enables multi‐step screening of millions of individual B cells within a span of two days, significantly accelerating the discovery of antibodies.

### 2.2 Fluorescent Halo‐Based Screening of Antigen Specific ASCs

**Figure 2:**
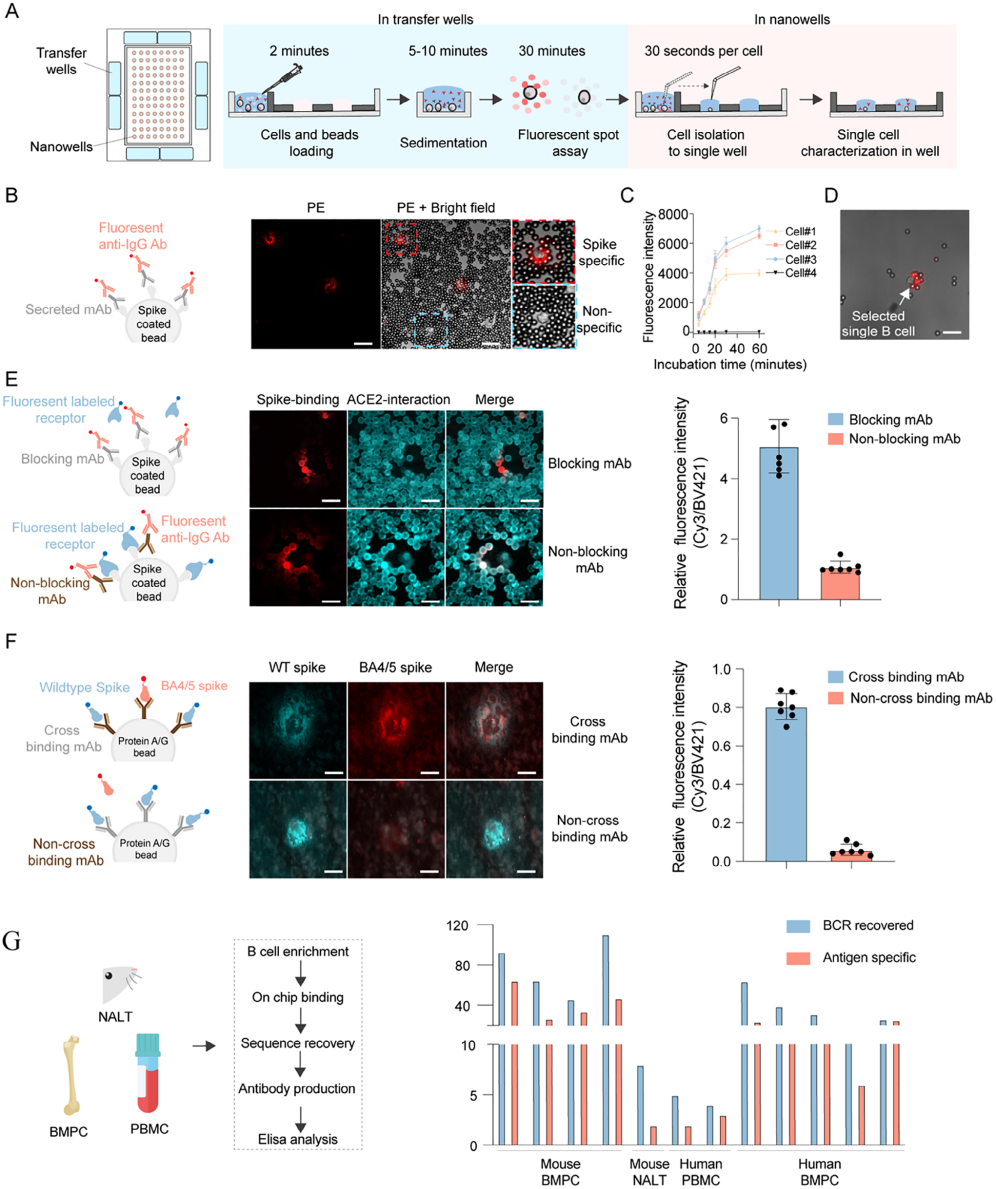
Beads‐based assay for screening and characterizing antigen‐specific ASCs on the MoSMAR‐chip. A) The overall workflow with indicated action and estimated time involved. Loading the mixture of enriched B cells, spike‐coated beads, and Fc‐specific fluorescent antibodies onto the MoSMAR‐chip transfer wells. Loaded mixture requires sedimentation and incubation before antigen‐specific B cells were visualized, picked by a robotic hand, and transferred to the microwells for further phenotyping and genotyping. Each microwell contains a single ASC. B) mAbs captured by spike‐coated beads are detected by PE‐labeled secondary antibody. Scale bar: 100 µm. C) Dynamic changes in fluorescence intensity of beads (*n* = 22) surrounding the spike‐specific cells over time, represented as mean ± SD. D) Representative image of a selected spike‐specific single B cell surrounded by the PE‐labelled beads in a microwell. Scale bar: 100 µm. E) Competition between secreted mAbs and ACE2 for binding to spike‐coated beads. Antibody binding spike was detected by Cy3 while hACE2 binding to spike was detected by BV421. The top panel illustrates an ACE2‐blocking antibody (Cy3‐positive and BV421‐negative) while the bottom panel illustrates an ACE2‐nonblocking antibody (Cy3‐positive and BV421‐positive). Right: Relative fluorescence intensity of beads (*n* = 14) for ACE2‐blocking and non‐blocking antibodies, represented as mean ± SD. Scale bar: 50 µm. F) Captured mAbs by protein A/G beads and their cross‐binding between wildtype and BA.4/5. Binding to wildtype spike was detected by fluorescence PE while to BA.4/5 by fluorescence BV421. The top panel illustrates cross‐binding (PE‐positive and BV421‐positive) while the bottom non‐cross binding (PE‐positive and BV421‐negative). Right: Relative fluorescence intensity of beads (*n* = 14) for cross‐binding and non‐cross‐binding antibody, represented as mean ± SD. Scale bar: 10 µm. G) Assessment of the method with various types of samples from mice and humans. Left: Enriched B cells analyzed for binding on MoSMAR‐chip, followed by BCR sequence recovery by RT‐PCR, mAbs production, and evaluation by ELISA. Right: Comparison between the number of BCR‐recovered and antigen‐specific B cells validated by ELISA.

We utilized the trimeric spike protein of SARS‐CoV‐2 as an antigen for screening antigen‐specific B cells. ASCs were functionally screened in the transfer wells of a MoSMAR‐chip (**Figure** **2**A) by cell‐bead or cell‐cell co‐incubation method to achieve a high sensitivity in detecting antibodies (Figure S2A,E, Supporting Information). In this case, spike‐coated beads or reporter cells expressing membrane‐bound spike protein were used as a capture interface for antibodies secreted by ASCs. Initially, spike‐coated beads were mixed with cells and suspended in medium containing fluorescent secondary antibodies. The mixture was then added to transfer wells, with ≈2000 cells and 0.1 µg beads per well, optimizing the bead‐to‐cell ratio (Figure S2B, Supporting Information). After 30 min of incubation, a fluorescence microscope was used to visualize fluorescent halos around cells secreting spike‐specific antibodies, while no fluorescence signal was observed near cells producing nonspecific antibodies (Figure 2B). Continuous monitoring of the fluorescence intensity of beads surrounding ASCs revealed a plateau after 1 h, indicating that the antigen on the beads had been saturated by the antibodies secreted by the ASCs (Figure 2C). Subsequently, target B cells displaying fluorescent halos were selected and individually transferred into microwells at a rate of 30 s per cell using the SCDI (Figure 2D; Figure S2C and Video S1, Supporting Information). Using this novel fluorescence halo method, we could also screen B cells with the ability to block receptor binding. Specifically, human ACE2 (hACE2)‐blocking mAbs inhibited the binding of fluorescently labeled ACE2 protein to spike‐coated beads. In contrast, cells secreting antibodies that lacked receptor‐blocking capability did not reduce the fluorescence signals from the ACE2 protein binding to the beads (Figure 2E). Additionally, we assessed the cross‐binding ability of cells sorted into microwells (Figure 2F). Similarly, by loading protein A/G beads into the microwells to capture antibodies and incubating them with fluorescence‐labeled antigens, we were able to detect antibodies capable of cross‐binding to mutant antigens, as they induced multiple fluorescence signals from the beads. As shown in Figure 2F, co‐localization of cyan and red fluorescence to the bead around cells indicated the presence of a cross‐binding antibody to both wildtype spike and BA4/5 spike.

To further evaluate the accuracy and sensitivity of the MoSMAR‐chip system, we tested ASCs of 12 samples isolated from bone marrow, nasal mucosa, or peripheral blood of autoimmune patients or vaccinated mice (Figure 2G). Some of these samples were immediately thawed and tested upon resuscitation. The starting cell number varies from 5 × 10^3^ to 8 × 10^5^. Each antigen‐specific B cells were exported to single centrifuge tubes, and the heavy‐ and light‐chain genes from single B cells were sequenced and cloned into immunoglobulin expression vectors for mAbs production, and evaluated by enzyme‐linked immunosorbent assay (ELISA). The results indicated that bone marrow samples from spike immunized mice had the highest antigen‐positive cell ratio (antigen specific cells / total exported cells), with 42.9% to 70.2% of the cells producing spike‐specific antibodies, measured by ELISA. In contrast, nasal mucosa or frozen human peripheral blood samples contained substantially lower ratio of antigen‐specific ASCs, ranging from 2 to 12 cells per 100000 cells, depending on the sample source. Notably, the antigen‐specific cells obtained from nasal mucosa were of the IgA subtype (Figure S2D, Supporting Information), known to play a critical role in the mucosal immune system against viral infection.^[^ ^9^ ^]^ Additionally, antigen‐positive ratios in human bone marrow mononuclear cells (BMMC) were similar to those from mice, ranging from 37.5% to 89.2%. Overall, these results demonstrated the robustness of tMoSMAR‐chip system in identifying antigen‐specific B cells and obtaining corresponding antibody clones from various in vivo samples.

### 2.3 Efficient Memory B Cell Activation and Screening for Antibodies using Microwell Co‐Culture

**Figure 3:**
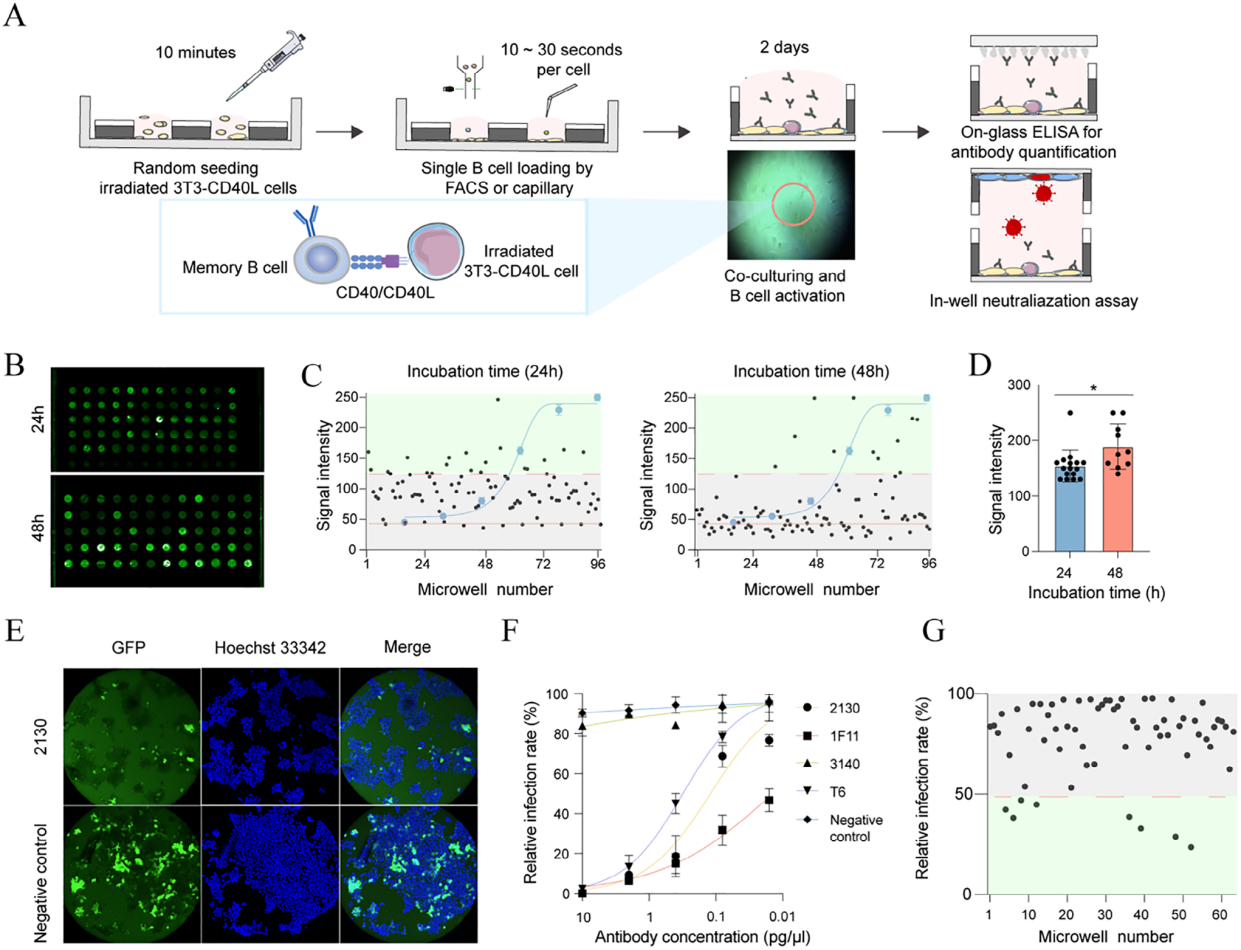
Co‐culture‐based assay for screening antigen‐specific MBC on the MoSMAR‐chip. A) The overall workflow with indicated action and estimated time involved. 3T3 cells expressing CD40L were seeded into microwells and irradiated before single memory B cells were loaded either through FACS or capillary deposition. Secreted antibodies were evaluated for binding through on‐glass ELISA and in‐well neutralization assay. B) Scanning image of the glass slides for on‐glass ELISA measuring antibody binding to anti‐human IgG at 24 h (top) and 48 h (bottom) post co‐culture. C) Signal intensity derived from each microwell after co‐culture for 24 h (left) and 48 h (right) compared with the standard curve, represented as mean ± SD. D) The average signal intensity among the positive B cells (*n* = 27) after co‐culture for 24 h (left) and 48 h (right), represented as mean ± SD. E) Representative images of GFP‐reporter virus‐infected 293T‐ACE2 cells in the presence (top) and absence of neutralizing mAb 2130 (bottom). F) Neutralization curves of various mAbs derived from in‐well neutralization assay, represented as mean ± SD. G) Relative infection rate of HEK 293T cells transfected with RBD‐binding mAb plasmids (*n* = 64) showing in‐well neutralizing activities analyzed by the MoSMAR‐chip.

The use of membrane‐bound BCRs as the single screening criteria for antigen‐specific memory B cells often leads to high false positive rates, posing challenges for subsequent analysis. To overcome this, we designed a workflow that enables co‐culture and activation of memory B cells with feeder cells within the microwells, followed by detection of secreted antibodies in the nanoliter droplets (**Figure** **3**A). Specifically, 3T3 cells expressing CD40L were seeded in microwells to serve as feeder cells, then the human IgG+ memory B cells enriched by FACS were distributed into microwells as single cells via the SCDI and cultured for 1–2 days. During this period, each memory B cell was continuously activated through the CD40L‐CD40 axis, leading to the secretion of IgG into the droplet within the microwell.

First, we performed ELISA on a glass slide in conjunction with the MoSMAR‐chip to confirm the sensitivity and the accuracy of this method. The ELISA standard curve obtained through the slide‐based ELISA was consistent with the results from the well‐plate ELISA (Figure S3A,B, Supporting Information). Next, the glass slides were divided into two regions, one was incubated with recombinant receptor binding domain (RBD) and the other with whole spike protein of SARS‐CoV‐2. Then, spike‐binding antibodies, either targeting RBD or non‐RBD regions, were added to the microwell array and docked with the slide, followed by detection using fluorescence‐labeled secondary antibody (Figure S3C, Supporting Information). Fluorescence signals appeared in both regions only when the slide was incubated with RBD‐targeting antibodies in the microwells, while non‐RBD‐targeting antibodies induced fluorescence exclusively in the region incubated with the spike protein, confirming the high accuracy and the consistency of the slide‐based ELISA (Figure S3D, Supporting Information).

To evaluate the dynamics of antibody production after stimulations, we divided MBCs into two experimental groups, one group was stimulated with feeder cells for 24 h while the other for 48h. The glass slide coated with anti‐IgG protein was then docked to the MoSMAR‐chip, allowing it to contact the IgG‐containing droplet, followed by detection using the fluorescent secondary antibodies (Figure 3B). The results showed that 16 out of 96 microwells after 24 h and 12 out of 96 microwells after 48 h co‐culture contained detectable IgG in the droplets (Figure 3C). Although the number of activated memory B cells with detectable IgG was slightly higher in the 24 h co‐culture group, the fluorescence intensity was significantly higher in the 48 h group (Figure 3D), indicating a rapid dynamic of antibody production and detection by the MoSMAR‐chip system.

Next, we evaluated whether secreted antibodies were capable of neutralizing SARS‐CoV‐2 pseudoviruses. We added pseudoviruses to each microwell containing RBD‐binding or ‐nonbinding antibodies. Subsequently, we employed a cover chip seeded with 293T‐ACE2 cells to assess the impact of antibodies on the infectivity of the pseudoviruses. In agreement with assays performed in regular 96‐well plates, all 4 antibodies showed similar neutralizing ability against SARS‐Cov2 pseudoviruses (Figure 3E,F), demonstrating the feasibility in assessing the neutralizing activity of antibodies in small‐volume microwells. Next, we transfected antibody plasmids into 293T cells to simulate activated MBCs. From these transfected cells, 64 single cells were sorted into microwells and cultured for 4 days to ensure sufficient antibody secretion. Using a 50% reduction in infection rate compared to the control group as the criterion, cells from 8 microwells secreted sufficient antibodies to effectively neutralize the pseudovirus in the microwells, preventing infection of the 293T‐ACE2 cells (Figure 3G). Thus, our co‐culturing and screening method can activate memory B cells and precisely identify those secreted mAbs with neutralizing activity.

### 2.4 Development of MoSMAR‐seq for Addressable Single B Cell Genotyping

**Figure 4:**
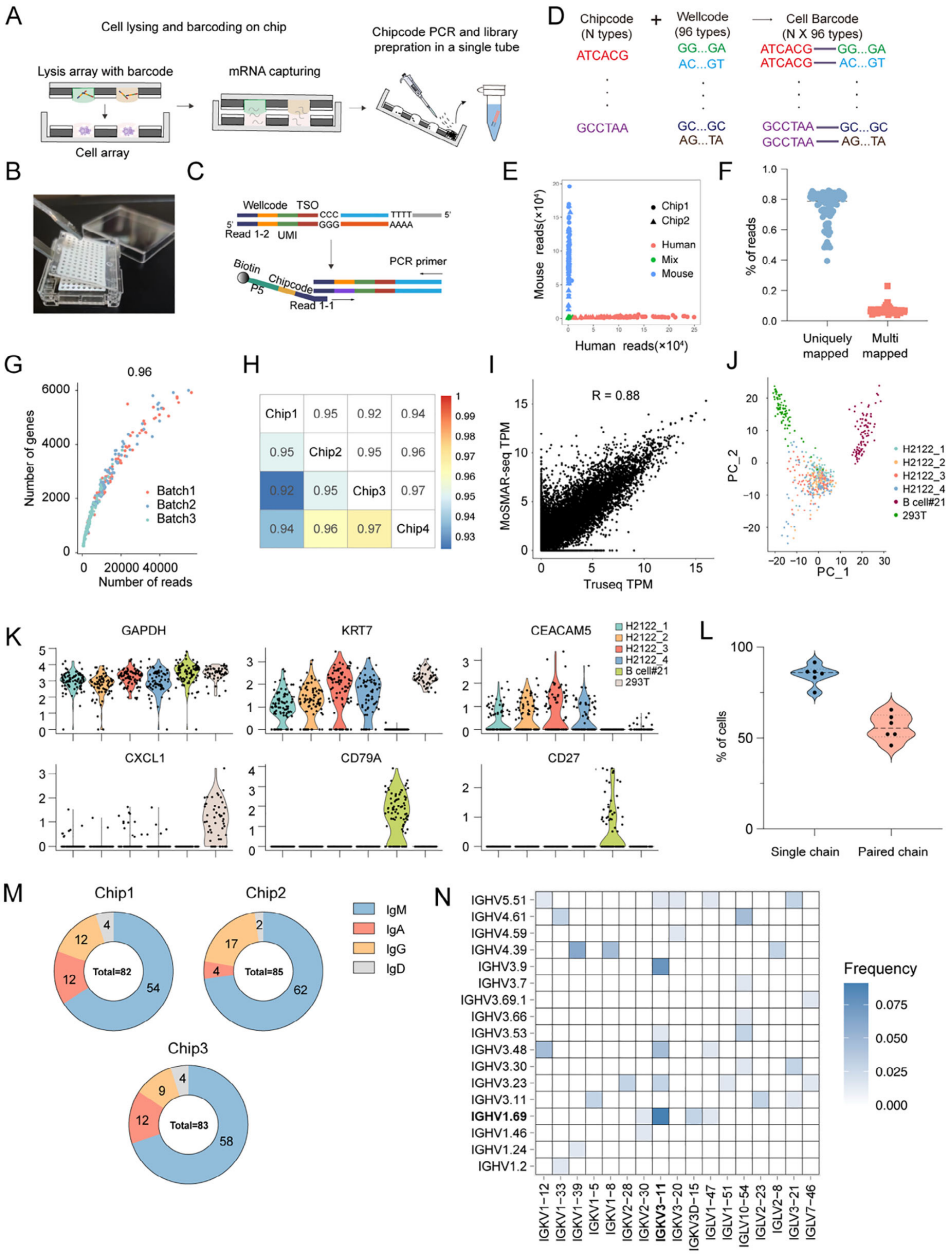
Performance characterization of the MoSMAR‐seq platform. A) Cell barcoding on MoSMAR‐chip. Cells were lysed in each single microwell and barcoded with TSO in droplets at 48 °C. Then, RT product was collected from the microarray and mixed, followed by chipcode ligation by PCR. B) Images showing the “spot‐cover” procedure of regent fast transferring. C) Schematic illustrating two‐step barcoding for each cell. D) N × 96 types of full‐length cell barcodes generated by the two‐step barcoding method. E) Mixed‐species experiment with 192 human (H2122) and 192 mouse (MC38) cells performed on MoSMAR‐chips. F) The percentage of uniquely mapped reads and multi‐mapped reads from 96 293T cells generated by MoSMAR‐seq. G) Numbers of reads and genes detected in single cells (*n* = 96) for three repeats of MoSMAR‐seq. H) Correlation coefficients plot between any two of sequenced chips (96 cells per chip). I) High correlation between the MoSMAR‐seq (*n* = 96) and the off‐chip TruSeq. J) PCA visualization of H2122 cells (*n* = 384), 293T cells (*n* = 96) and B cells (#21, *n* = 96) identified by performing unsupervised clustering analysis using differentially expressed genes (DEG) data before sample filtering. K) Violin plots showing the expression levels of the housekeeping gene (GAPDH) and signature genes for Epithelial Cells (H2122, 293T) and B cells (CD27). L) The percentage of B cells (*n* = 576) with at least one BCR chain or paired chains sequenced by MoSMAR‐seq, represented as mean ± SD. M) Constituent ratio of antibody types derived from sequenced MBCs (*n* = 250). N) Heatmap showing the paired of immunoglobulin heavy chains and light chains gene variable region segments of clonotypes from sequenced MBCs (*n* = 66).

To advance addressable genotyping at the single B cell level after screening for antibody binding and neutralizing activity, we developed a MoSMAR‐seq method for integrating gene expression analysis and immune repertoire profiling on the MoSMAR‐chip (**Figure** **4**A). Specifically, droplets containing a mixture of lysis buffer, template switching oligos (TSO) with microwell‐specific barcodes (wellcode, Figure S4A, Supporting Information), and reverse transcription (RT) buffer were precisely ejected onto the transfer cover clip, creating a highly ordered 12 × 8 droplet array for cell lysis and reverse transcription (lysis array) (Figure S4B, Supporting Information). The lysis array was docked onto the MoSMAR‐chip that containing up to 96 single B cells after screening for binding and neutralizing activities (cell array), as illustrated in Figure 4B. The B cells in the microwells were then lysed to release mRNA into the droplets, following with the incubation in a water bath at 48 °C for 1.5h. During reverse transcription, mRNA was captured by poly‐dT oligos with 3′ end extension containing a complementary sequence with TSOs and served as primer to synthesize the first‐strand cDNA. All synthesized cDNA from individual chips were collected by centrifugation and subjected to PCR for chipcode ligation (Figure 4C). The chipcodes embedding in the PCR primers were designed to further encode the cells on each MoSMAR‐chip, providing flexibility and scalability for subsequent sequencing while minimizing costs (Figure 4D; Figure S5, Supporting Information).

Next, we assessed the performance of the MoSMAR‐seq in terms of inter‐microwell contamination, library quality, sensitivity, repeatability, and accuracy. We distributed 192 H2122 (human lung adenocarcinoma) and 192 MC38 (murine colon adenocarcinoma) cells in alternative rows on the MoSMAR‐chip to assess the inter‐microwell contamination. As shown in Figure 4E, 190 of the 192 H2122 cells and 189 of the 192 MC38 cells were clearly separated according to their designated species. The sequencing data showed a unique mapping rate of 72.5% and a multi‐mapping rate of 4.9%, indicating accurate sequencing alignment, and high‐quality libraries with the majority of the data being informative and usable for downstream analysis (Figure 4F). To validate the sensitivity and repeatability of the MoSMAR‐seq, the experiments were repeated for three times using H2122 cells (96 cells each batch). The data showed that over 20000 reads corresponding to 2918 genes were detected from each cell, with a strong correlation across the three repeats (PCC, r = 0.96) (Figure 4G). Additionally, we cross‐compared data from 4 individual chips (96 cells each chip) to evaluate the impact of chipcode‐wellcode joint encoding, where high consistency (> 0.9) among the 4 chips was demonstrated (Figure 4H). Bulk RNA‐seq data were strongly correlated with populations constructed in silico from individual cells (96 cells) as shown in Figure 4I. Moreover, the presence of beads for functional screening did not lead to a decrease in pre‐amplification quality (Figure S4C, Supporting Information). To examine the capacity of the MoSMAR‐seq for comprehensive cellular deconvolution in complex multicellular systems, a mixture of single cells (*n* = 576) consisting of H2122, B cell and 293T was prepared. We used Seurat to disentangle the cell populations and visualize the data in PCA space (Figure 4J). The H2122, 293T, and B cells can be distinguished by the expression levels of *KRT4*, *CEACAM5*, *CXCL1*, *CD79A*, and *CD27*, respectively, while the housekeeping gene showed similar expression levels across all 3 cell types, demonstrating a high degree of consistency (Figure 4K). In addition to the gene expression analysis, we also evaluated the efficiency of immune repertoires of a B cell line. Nested PCR was performed to amplify BCR sequences from pre‐amplified cDNA followed by BCR library construction. After sequencing, 84.68% of 576 single B cells obtained either a heavy chain or a light chain and the cell proportion with paired chain was 55.88% (321 cells), comparable to other high‐throughput methods such as droplet‐based approaches (Figure 4L). Next, we evaluated the efficiency of recovering BCR sequences from co‐cultured MBCs in the presence of feeder cells. Single MBCs from PBMCs of a SARS‐CoV‐2 vaccinated human donor were sorted into MoSMAR microwell array, each well pre‐seeded with ≈200 3T3‐CD40L cells, followed by MoSMAR‐seq analysis (Figure S4D, Supporting Information). After library construction and sequencing, we successfully recovered single BCR chains from 85.41%, 92.7%, and 86.46% of the cells respectively from 3 individual chips (96 cells/chip). Isotype analyses of sequenced MBCs revealed consistent proportions of heavy chain isotypes across the 3 independent experiments (Figure 4M), with IgM being the dominant subtype (ranging from 65.83%–69.88%). Filtering out B cells without full‐length IGH or IGL chains reduced the proportion of cells with paired full‐length heavy and light chains to 22.92% (66/288), potentially due to the substantial amounts of RNA from feeder cells interfering with the reverse transcription of BCR sequences (Figure S4E, Supporting Information). Repertoire analysis of 66 cells with full‐length BCR sequences revealed that IGHV1‐69/IGKV3‐11, IGHV3‐9/IGKV3‐11, and IGHV4‐39/IGKV1‐39 as the top 3 most frequently paired heavy and light chain clonotypes (Figure 4N). Notably, IGHV1‐69/IGKV3‐11, the most enriched clonotype in our data, was also reported as the most commonly used V gene pair among the S2 antibodies in a previous study.^[^ ^10^ ^]^ The V and J gene combinations for both heavy and light chains, as well as the characteristics of the CDRH3 sequences of the sequenced MBCs, are detailed in Figure S4F–I (Supporting Information).

In conclusion, these results demonstrated the capacity of the MoSMAR‐seq for characterization of both transcriptome and immune repertoire at the single B cell level following screening for the binding and neutralizing activity of secreted antibodies in the supernatant of cell co‐culture.

### 2.5 Evaluating Long‐Term Antibody Response Induced by SARS‐CoV‐2 Vaccine by MoSMAR‐Chip

**Figure 5:**
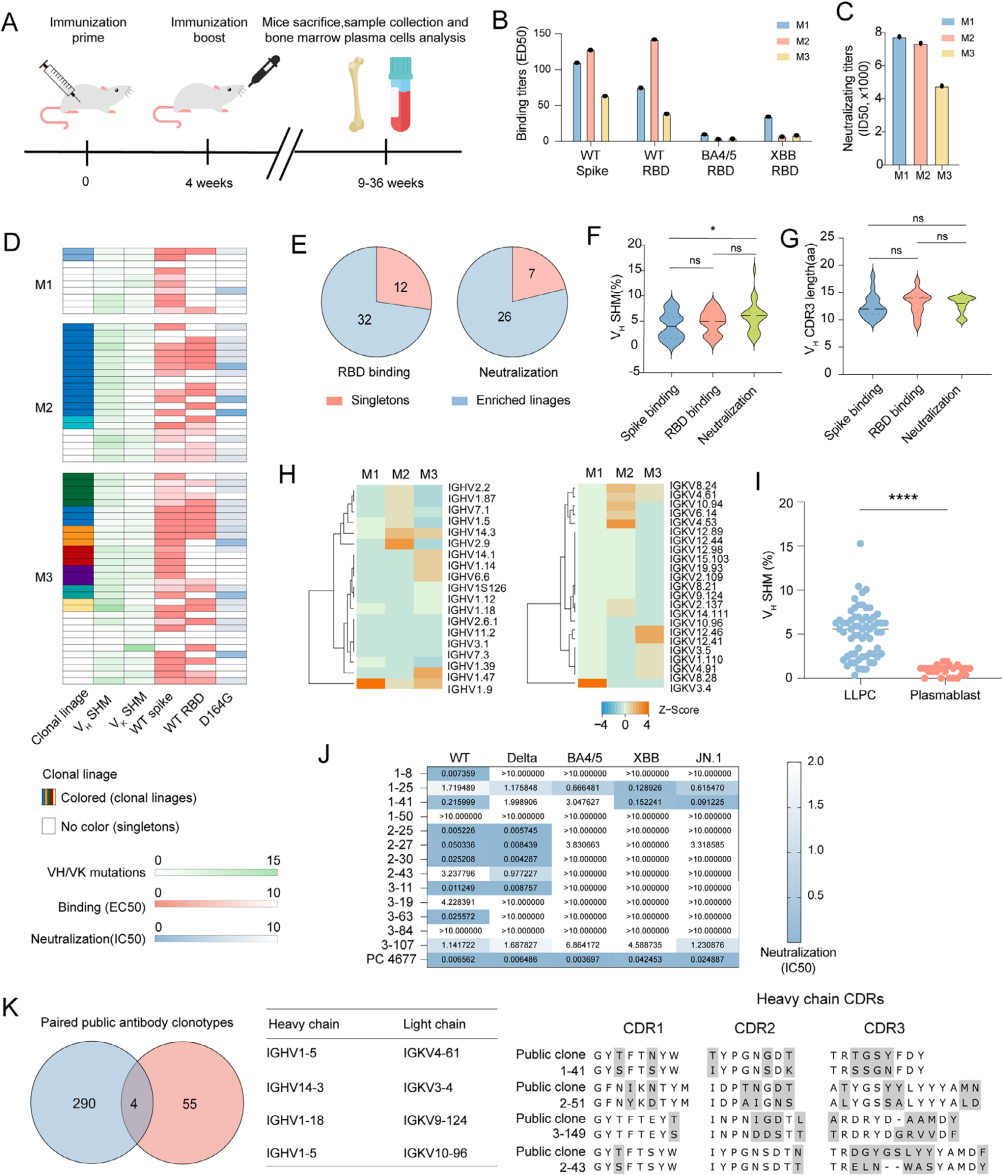
Evaluation of LLPC‐derived mAbs from immunized mice analyzed by MoSMAR. A) Experimental overview of mice immunization and sampling procedure. B) Serum binding titers against SARS‐CoV‐2 WT spike or RBD among 3 mice. C) Serum neutralizing titers against WT D614G pseudovirus among 3 mice. D) Clonality, degree of somatic hyper mutation, corresponding IC50 and EC50 among 65 monoclonal antibodies from 3 vaccinated mice ASCs. The EC50 was measured against WT S and RBD in the ELISA assay and the IC50 was measured against SARS‐CoV‐2 WT D614G pseudovirus. E) The number of mAbs with clonal expansion or singletons of RBD binding (left) and neutralization (Right). F) Violin plot showing somatic hypermutation of heavy chain from spike binding, RBD binding, or neutralization groups, represented as mean ± SD. G) Violin plot showing CDR3 length of heavy chain from spike binding, RBD binding, or neutralization groups, represented as mean ± SD. H) Variable segment usage of heavy chain (left) and light chain (right) across 3 mice. I) Compare of somatic hyper mutation degree of V_H_ of all synthesized mAbs from LLPC (*n* = 65) and plasmablast cells (*n* = 26). Data of plasmablast was adapted from Alsoussi et al., 2020. J) Neutralizing activity against WT D614G, Delta, BA.4/5, XBB and JN.1 SARS‐CoV‐2 pseudoviruses (IC50, µg/ml) of 13 randomly selected mAbs. K) Public antibody clonotypes shared by MoSMAR‐seq identified sequences (red) and previously published SARS‐CoV‐2 antibody sequences from the CoV‐AbDab database (blue), their germline usage(middle) and amino acid sequence of CDRs (right). Public data was retrieved from https://opig.stats.ox.ac.uk/webapps/covabdab/ (accessed on January 23, 2024).

Long‐lived plasma cells (LLPC) in the bone marrow contribute substantially to the long‐term antibody immunity to foreign antigens by continuously secreting antibodies.^[^ ^11^ ^]^ We evaluated the ability of the MoSMAR‐chip in isolating and characterizing spike‐specific antibodies in the bone marrow of SARS‐CoV‐2 vaccinated mice. All 3 experimental mice (labeled as M1, M2, and M3) were immunized twice with AdC68‐based vaccine^[^ ^12^ ^]^ in four‐week interval, and sacrificed after 9, 12, 36 weeks to collect and enrich bone‐marrow derived CD138*^+^* PCs (**Figure** **5**A). All 3 mice‐derived serum samples showed high binding titers to wildtype (WT) spike and RBD antigen, but reduced activity to BA4/5 and XBB variants (Figure 5B). Additionally, all samples demonstrated high neutralizing titers against the D164G variant (Figure 5C). From ≈1.1 × 10^6^ PCs in each mouse, 50, 52, and 220 antigen specific PCs were identified. After BCR profiling, the V gene germline usage was substantially different among the 3 mice. (Figure S6A, Supporting Information). Notably, VH1‐9 was the dominant germline (34.29%) in M2, while M3 exhibited a more diverse range of clonotypes. We successfully cloned, expressed, and purified 59 unique antibodies that demonstrated WT spike‐binding ability, with 44 of the 59 mAbs also binding the WT RBD (Figure 5D). Furthermore, SARS‐CoV‐2 pseudoviruses neutralizing assay revealed that 55.93% (33/59) of expressed mAbs were capable of neutralizing D164G pseudoviruses, an origin strain of SARS‐CoV‐2. Despite originating from the same germline and sharing close genetic relationships, these antibodies exhibited significant differences in neutralizing ability (Figure S6B, Supporting Information). Sequence analysis revealed that 62.7% of the isolated mAbs with spike binding ability belonged to expanded clonal lineages (Figure 5D), while a higher degree of clonal expansion was observed for the RBD binding group (72.7%) and neutralizing group (78.8%) (Figure 5E).

The analysis of antigen‐specific BCR sequences revealed that somatic hypermutation (SHM) was higher in the heavy chain of neutralizing antibodies than binding antibodies. (Figure 5F; Figure S6C, Supporting Information), perhaps due to higher levels of affinity maturation in the neutralizing group. No difference in CDR3 length was observed among 3 groups (Figure 5G; Figure S6D, Supporting Information). We also identified IGH1‐9 as a preferred germline usage among 3 mice, consistent with previous reports where a similar expanded clonotype was also noticed,^[^ ^4b^ ^]^ (Figure 5H). Interestingly, we found the SHM frequency was significantly higher from bone marrow‐derived PCs than those of reported plasmablasts response to SARS‐CoV‐2 spike^[^ ^13^ ^]^ (Figure 5I), suggesting PCs in the bone marrow may have undergone more cycles affinity maturation before they reside in the bone marrow.

Previous studies showed the presence of broadly neutralizing antibodies in vaccinated animals and humans by various vaccine modalities.^[^ ^14^ ^]^ Next, we tested whether the MoSMAR‐chip could allow us to isolate antibodies with similar potency and breadth. 13 clones were randomly selected from 59 mAbs and neutralizing assay was performed. As a result, 8 clones were able to neutralize Delta variants as expected, 4 clones were able to recognize BA4/5 variants, and 3 clones were capable of binding to XBB variants. To our surprise, 4 clones could even neutralize the JN.1 variant, which is the global dominant strain up to date^[^ ^15^ ^]^ (Figure 5J). Of tested mAbs, clone 1–25, 1–41, and 3–107, exhibited the broadest neutralizing abilities against all SARS‐CoV‐2 variants tested including JN.1, highlighting the robustness of the MoSMAR‐chip in isolating antigen‐specific antibodies in bone marrows.

Comparisons in clonotype with those in the public database (CoV‐AbDab^[^ ^16^ ^]^) revealed that antibodies from all immunized mice had similar clonotype and distribution (Figure S6E, Supporting Information). Despite differences in their CDR3 sequences, the heavy and light chains of 4 antibodies we isolated belonged to the same clonotype in the database (Figure 5K; Figure S6F, Supporting Information). WS6, which shares the same clonotype as 2–43, has been identified as an antibody capable of broadly binding to various viruses, including SARS‐CoV‐1, RaTG13, and Pangolin‐GD.^[^ ^17^ ^]^ Using AlphaFold3 to predict antibody‐antigen complex binding demonstrated that 2–43 binds to the I332 to T345 of the RBD region of spike protein^[^ ^18^ ^]^ (Figure S6G, Supporting Information). Sequence analysis of various viral variants revealed that the I332 to T345 region of RBD is conserved in early variants up to Delta, but became highly variable thereafter BA.4/5, XBB, and JN1 variants^[^ ^19^ ^]^ (Figure S6H, Supporting Information). This mutation pattern explains why the antibody 2–43 is ineffective in neutralizing key variants like BA.4/5 (Figure 5J). Overall, these results showed that the MoSMAR‐chip can be used to isolate antigen‐specific long‐lived plasma cells in the bone marrow, providing additional advantage over the traditional methods that rely on the surface expression of BCR.

### 2.6 Investigating Transcriptome Features of Antigen‐Specific Plasma Cells

**Figure 6:**
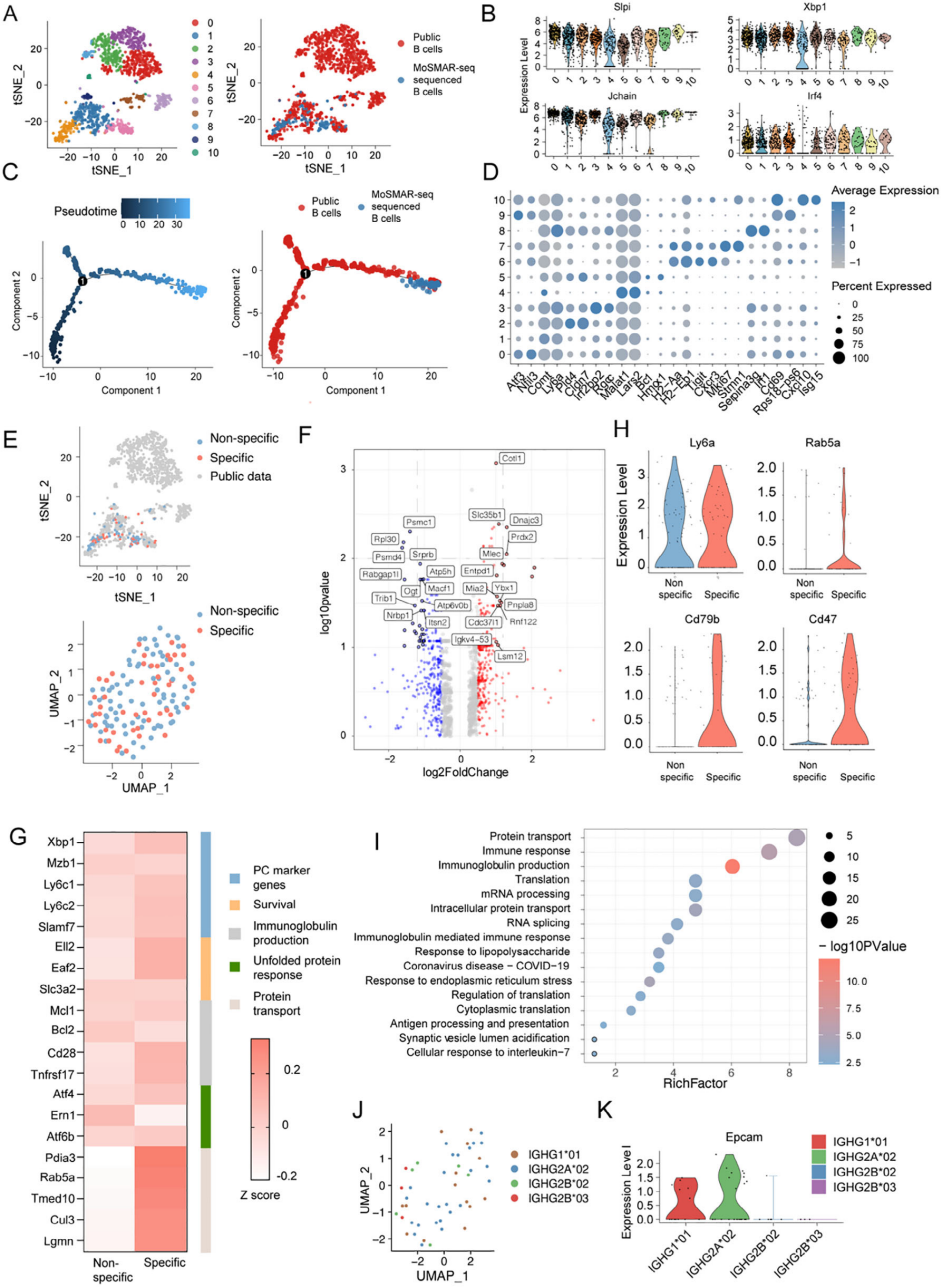
Investigating transcriptional features of SARS‐CoV‐2 spike‐specific LLPCs. A) Unbiased TSNE clustering analysis of 134 LLPCs from M3 with publicly available bone marrow plasma cells (*n* = 1234) from mice immunized with hIL‐4Rα antigen. B) Expression of known PC signature genes. C) Pseudotime trajectory analysis of total ASCs. D) Dot plots of selected gene expression, with the color intensity indicating expression level and the dot size indicating percentage of expressing cells in the PC clusters. E) TSNE and UMAP identified clusters of PCs based on SARS‐CoV‐2 spike specificity. F) Differential gene expression between antigen‐specific (*n* = 50) and non‐specific (*n* = 84) LLPCs. G) Heatmap showing the average normalized expression of B cell function‐related genes between antigen‐specific and non‐specific LLPCs. H) Violin plots of selected surface marker expression of antigen‐specific and non‐specific LLPCs. I) GO and KEGG enrichment analysis of the DEGs between the antigen‐specific and non‐specific LLPCs. J) SARS‐CoV‐2‐binding B‐cell isotypes are shown among sorted cells. K) Expression level of *Epcam* between different isotypes among sorted cells.

After data filtering, 60.9% of the cells from M3 (134/220) yielded reliable transcriptome sequencing results. Next, we integrated the 134 sequenced cells, of which 37.31% (50/134) were confirmed antigen‐specific (36 weeks post‐immunization) with single‐cell sequencing data derived from the mouse bone marrow plasma cells (*n* = 1234) in a public database. After using Harmony to remove batch effects,^[^ ^20^ ^]^ we identified 11 clusters of the bone marrow plasma cells. Interestingly, the cells analyzed by the MoSMAR‐seq primarily belonged to C1, C4, and C5 (**Figure** **6**A). All clusters highly expressed plasma cell‐related markers such as *Slpi* and *Xbp1*, suggesting their active state for producing immunoglobins (Figure 6B). The pseudotime analysis revealed distinct developmental pathways, with our identified plasma cell subpopulations positioned along these trajectories (Figure 6C; Figure S7A, Supporting Information). Notably, the subpopulations identified through the MoSMAR‐seq were located at the terminal branches of the pseudotime trajectory. Different from C6 and C7 cells, our identified cells showed reduced expression of *Mki67* and major histocompatibility complex (MHC) class II genes, signatures for newly minted ASCs^[^ ^21^ ^]^ (Figure 6D; Figure S7B,C, Supporting Information). Together, these results suggested that the MoSMAR‐seq identified cells with a long lifespan, indicating that they might be LLPCs. Since we have analyzed these LLPCs with positive antibody secretion in the aspect of immune repertoires, we further determined whether transcriptomic features could provide insights to differentiate antigen specific cells from non‐specific ones. First, we visualized 134 LLPCs identified in t‐SNE or uniform manifold approximation projection (UMAP) with or without integrating public data (Figure 6E). Although both subsets of sorted ASCs showed overlapped positions in unsupervised clustering, differential gene expression analysis identified coactosin‐like 1 (*Cotl1*) as an upregulated gene in antigen specific cells, which was reported to be critical for BCR‐induced actin remodeling and for T cell responses to APC‐bound antigens.^[^ ^22^ ^]^ Intersectin 2 (*Itsn2*), which was required for effective anti‐viral B cell immunity,^[^ ^23^ ^]^ was identified as a downregulated gene in antigen specific cells (Figure 6F). Consistent with previous studies, expression levels of features related to survival, immunoglobulin production, unfolded protein response and markers of PCs were relatively identical in both groups, whereas genes related to protein transport were significantly upregulated in the antigen specific group^[^ ^4a^ ^]^ (Figure 6G). In addition, B cell related surface markers such as *Rab5a*, *Cd79b*, and *Cd47* were upregulated in antigen specific cells as potential distinguished features, while *Ly6a*, which was reported to be highly expressed in LLPCs,^[^ ^24^ ^]^ remained stable (Figure 6H). Moreover, GO and KEGG enrichment indicated that pathways related to protein transport, immune response, immunoglobulin production and coronavirus disease were upregulated (Figure 6I), consistent with the function of LLPCs in SARS‐Cov‐2 vaccine immunized mice.

IgG1 and IgG3 are the dominant antibody isotypes elicited specifically against the virus spike protein and RBD after infection.^[^ ^25^ ^]^ In our study, 76% of identified antibodies (38/50) from M3 belong to IGHG2A*02 and IGHG1*01 with clonal expansion (Figure S7E, Supporting Information). Although the gene expression profiles were identical between expanded and nonexpanded groups (Figure 6J; Figure S7D, Supporting Information), the expression level of *Epcam* was upregulated in expanded clonotypes (*p* = 0.11), which was previously reported as features of antigen‐specific LLPCs^[^ ^24^ ^]^ (Figure 6K). Studies have demonstrated that LLPCs in older mice (16–36 weeks) tended to cluster and exclude newly generated PCs, resulting in a decrease of clonal diversity.^[^ ^26^ ^]^ This raised the question of whether LLPCs also interact with and reshape their niche to promote better clustering, as evidence suggests that PCs produce cytokines such as IL‐17, IL‐10, and IL‐35.^[^ ^27^ ^]^ To explore this, we conducted cell‐cell interaction analysis among various clonotypes (Figure S7F, Supporting Information). The ligand‐receptor results revealed a significant Cxcl12‐Cxcr4 axis between IGHG2A*02 and IGHG1*01 clonotypes (Figure S7G, Supporting Information), which had been identified as crucial for plasma cell homing and survival in NZB/W mice.^[^ ^28^ ^]^ Meanwhile, Il10‐Il10rb interaction was also identified among all 4 subtypes, suggesting the local myeloid lineage differentiation modulated by bone marrow‐derived PCs.

## 3 Discussion

Plasma cells and memory B cells collectively play essential roles in maintaining immunological memory following infection or vaccination for long‐term protection. Benefiting from the presence of specific antigen‐binding BCRs on MBCs’ surface, in recent years, there have been significant advancements in combining high‐throughput sorting and sequencing strategies for MBCs, although subsequent antibody validation processes often encounter false positives, posing challenges for subsequent analysis. Some sorting strategies involve stimulating memory B cells in culture dishes to convert them into ASCs, followed by microfluidic techniques for antibody secretion detection at the single‐cell level. However, this approach may lead to a loss of information on naturally expanded clonotypes. On the other hand, isolation of antigen‐specific ASCs has primarily been limited to sorting antigen‐specific plasmablasts (PBs), bulk sorting and RNA sequencing of total PC populations. While some elegant affinity matrix‐based methods have been described to capture antibody‐secreting plasma cells,^[^ ^29^ ^]^ these methods either have not been combined with high‐throughput sequencing of antigen‐specific plasma cells or are only capable of single round‐antigen‐specific screening.

To overcome these limitations and realize versatile identifying of interested B cells, we have developed a platform based on the MoSMAR‐chip, which is compatible with the screening of both ASCs and MBCs. Through the superhydrophobic microarray chip, we used antibody conjugated beads to perform multiplexed antibody characterization for ASCs. By simply using a pipette, we achieved a uniform distribution of single‐layer beads and cells in microfluidic chip transfer pools with appropriate apertures. The use of beads to separate cells greatly reduces the industrial difficulty and cost of processing microarray chips.^[^ ^5^ ^b]^ Additionally, this distribution method reduces sample loss compared to single‐cell segmentation using microdroplet methods.^[^ ^5^ ^d]^ Combined with our previously developed single‐cell distribution technology, rapid screening of antigen‐specific plasma cells is achieved. To further enhance efficiency, our next step involves integrating AI (Artificial intelligence) technology to identify the “halo” formed by the binding of antigens and antibodies, enabling the rapid localization and retrieval of antigen‐specific cells, despite the already achieved 5–10 × speed increase compared to microfluidic techniques such as nanopens.^[^ ^5a^ ^]^ For memory B cells, we performed a forward screen for antibody detection at a single‐cell level, which is achieved by co‐culturing memory B cells and feeder cells in a microwell. Owing to the increased detection sensitivity and the fast accumulation of secreted antibodies in the small culture volume of the MoSMAR‐chip, the detection time for MBCs has been shortened to 24 to 48 h, compared to 1–2 weeks required for culture in conventional plates.^[^ ^30^ ^]^ This acceleration has also increased the throughput and efficiency of monoclonal antibody isolation. In comparison to methods involving co‐stimulated culture followed by screening in 96‐well plates,^[^ ^31^ ^]^ we have retained the naturally expanded BCR clonotype information, which is significant for studying the evolution and differentiation of B cells post‐immunization or infection. Additionally, leveraging the feature of chip stacking, we can also achieve neutralization assay at a single cell level, even though this detection is for now binary.

In our previous work, we have successfully developed methods for phenotype‐genotype combined analysis at both multicellular and single‐cell levels, and extensively elaborated on the advantages of the chip platform.^[^ ^8^, ^32^ ^]^ In this study, by employing a chip stacking design, we have achieved nanoliter‐scale microfluidic reverse transcription in a semi‐open system, enabling the detection of 5′ mRNA sequencing, which facilitates the characterization of the variable regions of B cells. Meanwhile, this platform can also play an important role in identifying important TCRs during T cell differentiation and immune responses, such as monitoring continuously secreted cytokines by activated T cells (e.g., IFN‐γ). Compared to published single‐cell sequencing technologies based on conventional plates, such as smart‐seq3, we have achieved multi‐round characterization of single cells' phenotypes while also reducing the cost of addressable single‐cell sequencing to a similar level. MoSMAR‐seq is suitable for analyzing samples containing no more than 1–1000 cells, for example, functional immune cells, especially antigen‐specific ASCs and activated T cells secreting cytokines from organoids.

Studies have evaluated the efficacy of vaccines using mouse models,^[^ ^4b^ ^]^ yet research on LLPCs and their role in long‐term protection in mice post‐vaccination is rare due to their scarcity and lack of known surface markers for efficient cell isolation. We conducted immune repertoire analysis on LLPCs from three mice using our platform, with a focus on analyzing the transcriptome characteristics of M3 LLPCs after the longest post‐immunization period (36 weeks). We successfully isolated a considerable number of LLPCs from the bone marrow that exhibited binding with spike antigen at different affinities and broad‐spectrum neutralizing capabilities. Durable serum antibody titers are maintained by these cells, indicating their crucial role in long‐term immunity. At the single‐cell level, studying the unique characteristics of antibodies secreted by these cells can provide new insights into understanding long‐term immunity and enhancing immune efficiency.

In addition to serving as a tool for antibody discovery, our technology, when combined with transcriptomic approaches, can also provide fundamental insights into immunobiology. Many efforts have been made to identify biomarkers associated with antigen specificity for the rapid isolation of LLPCs.^[^ ^4^, ^20^, ^24^ ^]^ In this study, we found that spike‐specific LLPCs exhibit distinct gene expression pattern compared to non‐specific long‐lived plasma cells. *Cd47*, for instance, which acts as an immune checkpoint on macrophages to inhibit phagocytosis,^[^ ^33^ ^]^ is highly expressed in spike‐specific. This elevated expression may provide evidence that LLPCs can survive following vaccination.

## 4 Conclusion

In summary, we introduce the MoSMAR‐chip platform, a versatile, cost‐effective, high‐throughput tool that enables the identification, isolation, and functional analysis of antigen‐specific LLPCs and MBCs at the single‐cell level. Our results, demonstrated using SARS‐CoV‐2 immunized mice, highlight the platform's capability to identify and isolate neutralizing antibodies with broad‐spectrum activity, providing critical insights into long‐term immunity mechanisms. By bridging single‐cell functional analysis with immune repertoire and genomic profiling, this work significantly advances our understanding of B cell and plasma cell biology. We believe the MoSMAR‐chip platform holds strong potential for investigating immune responses in various clinical contexts, including vaccine development, autoimmune diseases, and T cell immune responses, thereby contributing to the development of targeted immunotherapies and precision medicine

## 5 Experimental Section

### Ethics Statement

This study received approval from the Research Ethics Committee of Beijing Youan Hospital, China (LL‐2020‐039‐K) and Shenzhen Third People's Hospital (2020‐084). The research was conducted in strict accordance with the rules and regulations of the Chinese Government for the protection of human subjects. The study subjects agreed and signed the written informed consent for the use of their blood samples in research. All animal experiments were carried out in strict compliance with the Guide for the Care and Use of Laboratory Animals of the People's Republic of China and approved by the Committee on the Ethics of Animal Experiments of Tsinghua University (22‐ZLQ2). Mouse immunization and characterization were conducted in the animal facility of Tsinghua University.

### Chip Manufacturing

A MoSMAR‐chip was fabricated by standard injection molding with polystyrene as previously described^[^ ^8^ ^]^ (Hochuen Technologies, Shenzhen, China). The chip surface was plasma‐treated to ensure high cell viability. Superhydrophobic paint was prepared following Lu's protocol.^[^ ^34^ ^]^ Briefly, 1 g of 1H, 1H, 2H, 2H‐perfluorooctyltriethoxysilane (Sigma‐Aldrich, St. Louis, USA) was added into 99 g of absolute ethanol and mechanically stirred for 2 h. Then, 6 g of titanium oxide (TiO_2_) nanoparticles (≈60 to 200 nm) (Sigma‐Aldrich, St. Louis, USA) and 6 g of P25 TiO_2_ (≈21 nm) (Degussa, Essen, Germany) were added into the solution to make a paint‐like suspension. The paint was then pipetted onto the top surface of the microwell array chip and air‐dried completely. The flexibility of the MoSMAR‐chip, along with layers that possess both hydrophobic and hydrophilic properties, enables complex manipulations within nano‐scale droplets (Figure S1, Supporting Information).

### HEK 293T Cells Transfection with Antibody Expression Vectors

In the in vitro validation of the ASCs functional analysis method, HEK 293T cells secreting antibodies were used to mimic ASCs. 1 µg of heavy chain plasmids, 1 µg of light chain plasmids, and 8 µl of polyethyleneimine (PEI, Sigma, USA) were added into 100 µl of Opti‐MEM transfection medium (Life Technologies, USA). After a 15‐min incubation, the mixture was added to HEK 293T cells per well in a 12‐well cell culture plate. Cells were re‐suspended using DEME medium after cultured at 37 °C for another 24 h.

### Recombinant Protein Expression and Purification

A recombinant wildtype spike trimer, RBD, and human receptor ACE2 peptidase domain protein were produced by transfection of expression vectors into 293F cells using polyethyleneimine (Sigma, USA). The recombinant spike and RBD proteins contained the C‐terminal Strep‐tagII (8 amino acids, WSHPQFEK), and the human ACE2 protein contained the C‐terminal 8×his tag. 600 µg of expression plasmids in 25 ml of Opti‐MEM transfection medium, and 2.4 ml of PEI in 25 ml of Opti‐MEM was mixed and incubated for 15 min. The mixture was added to 600 ml of HEK 293F cells at a density of 2.2 × 10^6^ cells ml^−1^. After 4 days, the supernatant containing secreted protein was collected, filtered using 0.45 µm filters, captured using Strep‐Tactin resin (IBA‐lifesciences, Germany) or Ni‐NTA Agarose (QIAGEN, Germany), and purified by SEC on a Superdex 200 Increase 10/300 GL column (GE Healthcare, USA). The production of biotinylated spike protein was achieved by the expression vectors of recombinant spike contained the C‐terminal 8×his tag and AviTag (15 amino acids, GLNDIFEAQKIEWHE) together with BirA expression plasmids that were co‐transfected into HEK 293F cells with 100 µm biotin, according to a previous paper.^[^ ^35^ ^]^ After a 4‐day culture, the biotinylated spike protein was collected and purified by procedures as described above. The purified biotinylated protein was incubated with PE Streptavidin or BV421 Streptavidin (High Concentration) (Biolegend, USA) for 20 min at room temperature, and then incubated with 5 µm biotin for 3 min at room temperature.

### Identifying Antigen‐Specific ASCs by MoSMAR‐Chip

Fifty microliters of streptavidin beads (Spherotech, USA) were incubated with 5 µl biotinylated antigen protein(0.5 mg ml^−1^) at 4 °C for 30 min. Then the beads were centrifuged, re‐suspended, and washed three times with PBS. Finally, the beads were re‐suspended in 50 µl of RPMI 1640 medium and stored at 4 °C until used. Coated beads were mixed with 8000 cells to a final volume of 70 µl with 1.2 µl of fluorescent antibody (Anti‐mouse IgG‐AF561, Thermo fisher, USA). The mixture was loaded into transfer wells of the MoSMAR‐chip (20 µl per well). A full chip was sealed with parafilm and incubated at 37 °C for 30 min and imaged with a confocal microscopy. Positive cells showing fluorescence halos were transferred into microwell with the single‐cell distribution instrument (SCDI) as described.^[^ ^8^ ^]^

### Receptor Blocking Assay

Fifty microliters of antigen‐coated beads were mixed with 8000 cells to a final volume of 70 and 1.2 µl of fluorescent antibody was added (Anti‐mouse IgG‐AF561, Thermo fisher, USA). The mixture was loaded into transfer wells of a MoSMAR‐chip (20 µL well^−1^). The full chip was sealed with parafilm and incubated at 37 °C for 30 min. Then the chip was unsealed and 10 µl of HIS tagged hACE2 protein(0.5 mg ml^−1^), 1 µl of fluorescent antibody Anti‐His tag, Thermo fisher, USA) were added into each well. The chip was sealed again and incubated at 37 °C for 30 min and imaged with a confocal microscopy.

### Cross‐Reactive Assay

Fifty microliters of protein A/G magbeads (Beaver, China) were at first mixed with 5 µl of fluorescence‐tagged antigens. Then, ≈250 nl of the mixture was added into each microwell by droplet dragging on superhydrophobic surface. The full chip was sealed with parafilm and incubated at 37 °C for 30 min and imaged with confocal microscopy.

### Antibody Expression and Purification of mAbs

The standard smart‐seq2 protocol was used to perform reverse transcription and cDNA amplification for single plasma cells.^[^ ^36^ ^]^ The IgG heavy and light chain variable genes were amplified using a 2‐step nest PCR and cloned into antibody expression vectors. Antibodies were produced by co‐transfection of the heavy and light chain expression plasmids into Expi293 cells using polyethyleneimine (PEI, Sigma, USA). Specifically, 25 µg of heavy chain plasmids, 25 µg of light chain plasmids and 200 µl of PEI were added into 1 ml of Opti‐MEM transfection medium (Life Technologies, USA). After a 15‐min incubation, the mixture was added to 25 ml of Expi293 cells at an approximate density of 3 × 10^6^ cell ml^−1^. After 4 days, the supernatant was collected, and secreted antibodies were purified using Protein A magnetic beads (GenScript, USA). The concentration of antibodies was determined by nanodrop 2000 Spectrophotometer (Thermo Scientific, USA). Sequences used in this section are listed in Table S1 (Supporting Information).

### Feeder Cell Preparation

3T3‐CD40L cells were used as feeder cells and passaged using DMEM at a ratio of 1:10. Two days later, cells from five dishes of 3T3‐CD40L were digested with trypsin for ≈10 min. The reaction was terminated with DMEM containing 10% FBS, and cells were collected into a 50 ml centrifuge tube and centrifuged at 800 rpm for 5 min to remove the supernatant. The cells were resuspended in 50 ml of 10% FBS PS DMEM to a density of 9 × 10^5^ cells ml^−1^. After sealing with a cover and wrapping with sealing film, the cells were irradiated with a dose of 4.5 Gy.

### Isolation of Memory B Cells from Donors Immunized with SARS‐Cov‐2 Vaccine

PBMCs were aliquoted into 1.5 ml tubes according to the designed staining panel and centrifuged at 1500 rpm, 4 °C for 5 min. The cells were then resuspended in PBS (2% FBS) with the appropriate volume, kept in the dark, and the corresponding antibodies were added. The mixture was incubated at 4 °C for 1 h. After incubation, the cells were centrifuged at 1500 rpm, 4 °C for 5 min, and resuspended in 1 ml of PBS (2% FBS). This wash step was repeated once more, and the cells were resuspended again in 1 ml of PBS (2% FBS), followed by centrifugation at 1500 rpm, 4 °C for 5 min. Finally, the cells were resuspended in 500 µl of PBS (2% FBS) and transferred to flow cytometry tubes equipped with filters. Antibodies used in this section were listed in Table S2 (Supporting Information).

### Identifying Antigen‐Specific Memory B Cells by MoSMAR‐Chip

Approximately 300 feeder cells were allocated into each microwell and cultured overnight. Isolated memory B cells were single‐sorted into each well and cultured at different timepoints. Glass‐slide ELISA were performed to identify antigen‐specific cells after stimulation. Briefly, goat anti‐human IgG‐UNLB (Biolegend, USA) was utilized as the antigen protein for capturing IgG proteins secreted by single cells, diluted in PBS at a 1:5 ratio, and coated onto epoxy‐modified slides, followed by overnight incubation at 4 °C. Subsequently, the slides were immersed in a 2% BSA solution for 30 min for blocking and washed with a PBS‐Tween solution (0.1% Tween‐20) on a shaker at 100 rpm for 10 min, repeated three times, followed by nitrogen drying to complete the antigen protein modification. MoSMAR‐chips were then prepared for protein detection, with the antigen‐modified slides aligned with the chip subunits and incubated in a 37 °C incubator for 40 min for antigen‐antibody hybridization. Post‐hybridization, slides were washed again with PBS‐Tween, dried with nitrogen, and PE‐labeled goat anti‐human IgG Fc secondary antibody was added to chip subunits. Alignment of the antibody‐captured slides with secondary antibody droplets in the chip subunits was followed by a further 40 min incubation at 37 °C to complete antibody‐secondary antibody hybridization. After hybridization with the fluorescent secondary antibody, slides were washed in PBS‐Tween, dried in nitrogen, and then subjected to scanning with a microarray chip scanner to obtain fluorescent signal images. ImageJ software was used for image signal quantification and statistical evaluation.

### In Well Neutralizing

A cell chip seeded with 200 of 293T‐ACE2 cells was prepared and cultured overnight to allow cell settling. A pseudotyped virus chip was prepared by loading 500 TU ml^−1^ of pseudotyped virus and 0.1–100 pg of mAbs into each well and incubated for 1 h. The cell chip was then aligned with a pseudotyped virus chip and incubated for 24 h at 37 °C. The cell chip was then scanned on an Olympus IX83 inverted fluorescence microscope and the infection rate was quantified after imaging.

### Wellcode Design and Lysis Array Preparation

Wellcode was designed in the middle region of template switching oligo (TSO) based on hamming codes for minimal distance properties^[^ ^37^ ^]^ (Figure S4a and Table S3, Supporting Information). To prepare a lysis array, TSOs, reverse transcription (RT) mix and lysis mix were all transferred by Echo to microwells with a volume of 300 nl. The loaded transfer coverslip was then stored at 4 °C until use. Reagents used for RT mix and lysis mix are listed in Table S4 (Supporting Information).

### Cell Lysis, Reverse Transcription and Template Switching

Microarray with target cells were first quickly washed with nuclease‐free water to remove medium and fetal bovine serum (FBS). A prepared wellcode array was aligned with a cell array immediately. Cells were lysed and released mRNA was captured by capture oligos in the lysis buffer. Then, the chip was sealed in a self‐seal bag and immersed in water bath at 48 °C. After 90 min of incubation, both chips containing RT product were washed by minor oil. cDNA product with oil were all collected into a 50 ml tube using an adaptor and centrifuged for 1 min. The water layer was recycled into a new 1.5 ml tube and purified by zymo DNA purification kit (Zymo research, USA). cDNA was eluted in 12 µl of ddH_2_O and stored at −20 °C until used.

### Chipcode Ligation PCR

Sequences and regents used for chipcode PCR are listed in Table S5 (Supporting Information). A PCR mix containing 20 µl of 2× HotStart Readymix (Kapa Biosystems, USA), 4 µl of chipcode primer (10 µm) and 4 µL of reverse primer (10 µm) was added to 12 µl of purified cDNA. The PCR program was as follows: 95 °C for 3 min; then 4 cycles of: 98 °C for 20 s, 65 °C for 45 s, and 72 °C for 3 min; then 18 cycles of 98 °C for 20 s, 67 °C for 20 s and 72 °C for 3 min; then a final extension step of 5 min at 72 °C. The PCR products were purified using 0.6 × Agencourt AMPure XP beads (Beckman Coulter, USA) according to the manufacturer's instructions, and eluted into 50 µl of ddH_2_O.

### BCR Enrichment PCR

Sequences and regents used for BCR enrichment PCR are listed in Table S6 (Supporting Information). A PCR mix containing 10 µl of 2 × HotStart Readymix (Roche, Switzerland), 2 µl of BCR outer primer (10 µm) and 2 µl of P7 primer (10 µm) was added to 6 µl of post cDNA amplification samples. The PCR program was as follows: 95 °C for 3 min; then 10 cycles of: 98 °C for 20 s, 65 °C for 45 s, and 72 °C for 3 min; then a final extension step of 5 min at 72 °C. The PCR products were purified using 0.8× Agencourt AMPure XP beads (Beckman Coulter, USA) according to the manufacturer's instructions and eluted in 6 µL of ddH_2_O. The product was used to perform second round of enrichment PCR changing to the BCR inner primer listed in Table S6 (Supporting Information). The final product was purified using 0.8× Agencourt AMPure XP beads (Beckman Coulter, USA) according to the manufacturer's instructions and eluted in 50 µl of ddH_2_O.

### Library Construction and Sequencing

PCR products were fragmented by Covaris M220 (Covaris, USA) following the manufacturer's instruction. After DNA shearing, end repair, 5′ phosphorylation, dA‐tailing, adaptor ligation, size selection, and PCR enrichment were sequentially conducted using the NEBNext Ultra II DNA Library Prep Kit for Illumina (NEB, USA). Sequences used in this section are listed in Table S7 (Supporting Information). Final PCR products were purified using 0.8× Agencourt AMPure XP beads (Beckman Coulter, USA), and the library quality was assessed on the Agilent Bioanalyzer 4200. Paired‐end sequencing was performed on an Illumina NovaSeq 6000 sequencing system for RNA‐seq and V(D)J libraries. For RNA‐seq libraries, STARsolo (version 2.7.8a) was used to perform sample de‐multiplexing, alignment, filtering, and UMI counting.^[^ ^38^ ^]^ The human GRCh38 and mouse mm10 genome assembly and RefSeq gene model for humans and mice, respectively, were used for alignment. For V(D)J libraries, the TRUST4 software was used to perform sample de‐multiplexing, to *de novo* assemble read pairs into contigs, to align, and to annotate contigs against all V(D)J germline reference sequences. ^[^ ^39^ ^]^

### Mixed‐Species Experiment

H2122 and MC38 cells were single‐sorted into the microwells of a single MoSMAR‐chip in an alternate line format. Cells were cultured overnight, followed by cell lysising and reverse transcription in well. cDNA pre‐amplification and library construction were performed as described above.

### Comparison of MoSMAR‐seq to Bulk RNA‐Seq

In the MoSMAR‐seq, 96 H2122 cells were allocated into each microwell and a pooled library was constructed as described above. In the meantime, 10^5^ of H2122 cells from the same batch were collected to extract total RNA using a RNeasy column (Qiagen, Germany) and a sequencing library was prepared from the total RNA using the Illumina TruSeq poly(A) + RNA‐Seq library construction kit (NEB, USA) according to the manufacturer's instruction.

### Mouse Immunization and Sample Collection

Female BALB/c mice aged 6–8 weeks were intramuscularly vaccinated and intranasally boosted with two doses comprising 10^10^ vp of AdC68‐based vaccines encoding the spike protein from wild‐type SARS‐CoV‐2 as previously reported with 4 weeks interval.^[^ ^12^ ^]^ During 9–36 weeks after initial immunization, animals were sacrificed to collect serum and bone marrow samples. The serum was heat‐inactivated at 56 °C for 30 min and analyzed for SARS‐CoV‐2 specific binding and neutralizing antibodies.

### Plasma Cells Isolation and Enrichment

Bone marrow cells were isolated from mice tibia and femur, and plasma cells were isolated using the mouse CD138+ plasma cells isolation kit (Miltenyi Biotec, Germany). Specifically, single‐cell suspension from mice bone marrow was centrifuged and re‐suspended with a non‐plasma cell depletion cocktail, containing biotin‐conjugated anti‐mouse antibodies against CD49b (DX5) and CD45R (B220). After incubation for 10 min and wash with phosphate‐buffered saline containing 0.5% FBS buffer, cells were mixed and incubated with anti‐biotin microbeads for 15 min, applied to a LD column, and the flow‐through unlabeled cells were collected. The pre‐enriched cells were then centrifuged, re‐suspended, and incubated with CD138 microbeads for 15 min. To positively separate the magnetically labeled cells, the cell suspension was applied to a MS Column. After wash with buffer, the column was removed from the separator. The enriched plasma cells were collected by pipetting 1 ml of RPMI‐1640 medium onto the column, applying the plunger, and flushing out the labeled cells.

### ELISA Binding Measurement of Serum and Antibody

The serum samples from immunized mice or monoclonal antibodies were serially diluted and added to 96‐well plate pre‐coated with 100 ng well^−1^ SARS‐CoV‐2 Spike trimer or RBD produced in HEK 293F cells. The plates were incubated at 37 °C for 1 h, washed three times with a phosphate‐buffered saline containing 0.06% Tween 20 (PBST). Then, the plates were incubated with secondary horseradish peroxidase (HRP)‐conjugated antibody against mouse IgG or human IgG (1:4000, Promega, USA) at 37 °C for 1 h. The samples were washed three times with PBST before the substrate TMB (3′,3′,5′,5′, ‐tetramethyl benzidine) was added. The reaction was stopped by adding 1 m H_2_SO_4_, and absorbance at 450 and 655 nm was measured using a microplate reader (Bio‐Rad, USA). The ED50 value was calculated based on binding curves drawn in Prism 8.0 software (GraphPad Inc., USA).

### Pseudovirus Neutralizing Measurement of Serum and Antibody

The SARS‐CoV‐2 pseudovirus neutralizing activity of vaccinated mice serum and monoclonal antibodies was determined using assays as previously reported.^[^ ^40^ ^]^ HEK293T cells were co‐transfected with the HIV backbone expressing firefly luciferase (pNL43R‐E‐luciferase) and pcDNA3.1 (Invitrogen, USA) encoding the wildtype Wuhan‐Hu‐1 (GenBank: MN908947.3) and variants. After 72 h, the supernatant containing the pseudovirus was collected and stored at −80 °C. To measure the neutralizing activity, mice serum or antibodies were serially diluted three‐fold in the 96‐well cell culture plates, and SARS‐CoV‐2 pseudovirus was added. After incubation at 37 °C for 1 h, ≈1.5 × 10^4^ HEK293T‐hACE2 cells were added into the mixture and incubated further at 37 °C for 48 h. The relative light units were measured using Bright‐Glo Luciferase Assay Vector System (Promega, USA) by GloMax Discover microplate reader (Promega, USA). The ID50 values were calculated using Prism 8.0 (GraphPad Inc., USA).

### Statistical Analysis

Flow cytometry data were analyzed with FlowJo software. Image J was used for image signal processing and quantitative statistics. For single cell sequencing, all individual single‐cell samples with >300 transcripts were retained in the analysis. After the aforementioned quality control steps, the filtered single‐cell transcriptomic expression matrix obtained from the data was subjected to further analysis using Seurat^[^ ^41^ ^]^(version 4.2.0). Batch effect was removed using Harmony.^[^ ^42^ ^]^ The ligand‐receptor interaction‐based cell communication analysis was completed by CellCall^[^ ^43^ ^]^ (version 0.0.0.9000). Statistical tests were run as indicated in the individual figure legends using GraphPad Prism 7.02 software and R studio. Information related to the size of data was indicated in figure panels or figure legends. The data were presented as the means ± standard deviations, medians, or quartiles, as appropriate. Normally distributed variables were analyzed by Student's t‐tests. The significances were denoted as * and ns, (specifically, *P < 0.05, **P < 0.01, ***P < 0.001, ****P < 0.0001, P value lower than 0.05 is considered significantly different).

## Conflict of Interest

The authors declare no conflict of interest.

## Supporting information



Supporting Information

Supplemental Tables

Supplemental Video 1

## Data Availability

The data that support the findings of this study are available from the corresponding author upon reasonable request.
