# Supplementary material for: A Versatile High‐Throughput Single‐Cell Screening Platform for Profiling Antigen‐Specific Long‐Lived B Cells in Blood and Bone Marrow
Source: Adv Sci (Weinh). 2025 Apr 9;12(21):2414945. doi: 10.1002/advs.202414945 (PMC12140366; doi:10.1002/advs.202414945)
Supplement: Supplementary file 1 — Supporting Information [file ADVS-12-2414945-s003.docx]

Supporting Information

A versatile high-throughput single-cell screening platform for profiling antigen-speciﬁc long-lived B cells in blood and bone marrow

Tian Zhao, Yuqing Lei, Chang Liu, Dong Zhang, Kaiyi Li, Sisi Shan, Chenyu Li, Zimeng Wei, Yuhan Yang, Ting Zhang, Kai Sun, Haoran Sun, Linqi Zhang* and Peng Liu*


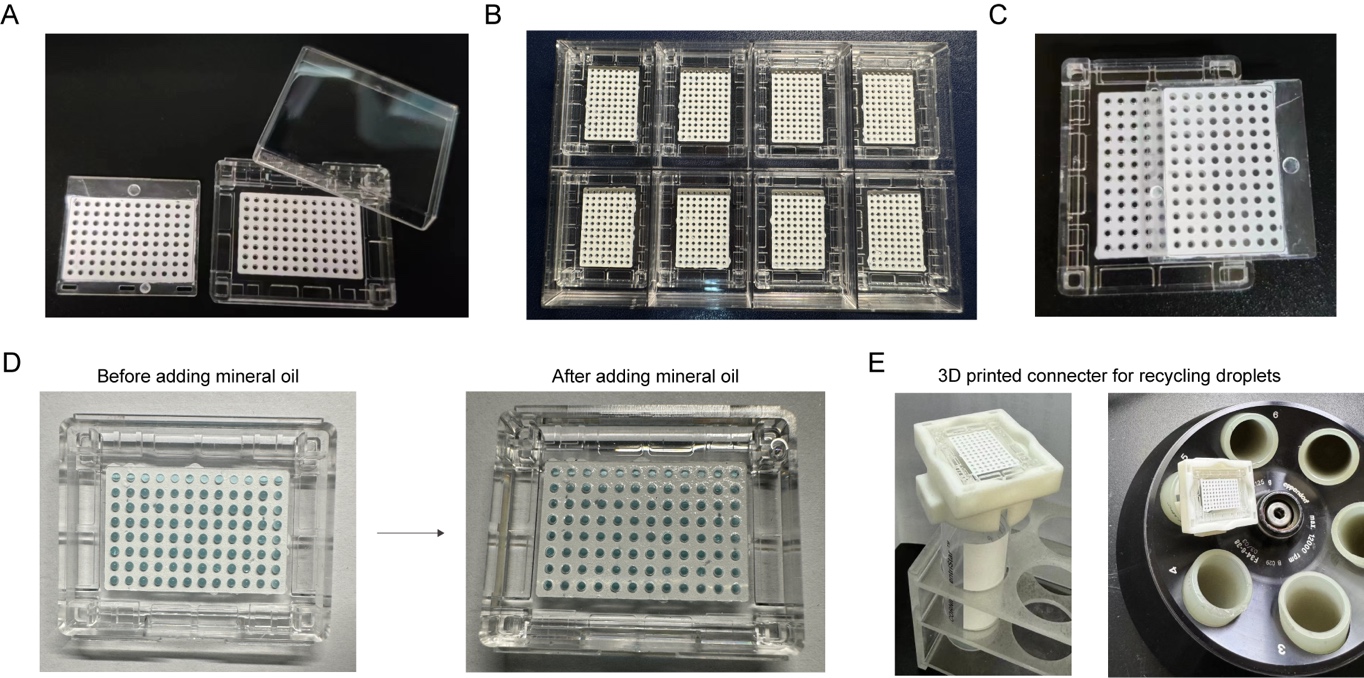


**Figure S1 Architecture and operation of MoSMAR-chip. (A)** to **(C)** photographs showing the structure and dimensions of the assembled MoSMAR-chip, the reaction chip, and the transfer coverslip. **(D)** The droplet morphology before (left) and after (right) the addition of mineral oil for droplet sealing. **(E)** 3D printed adaptor for quickly recycling droplets after bioassays.


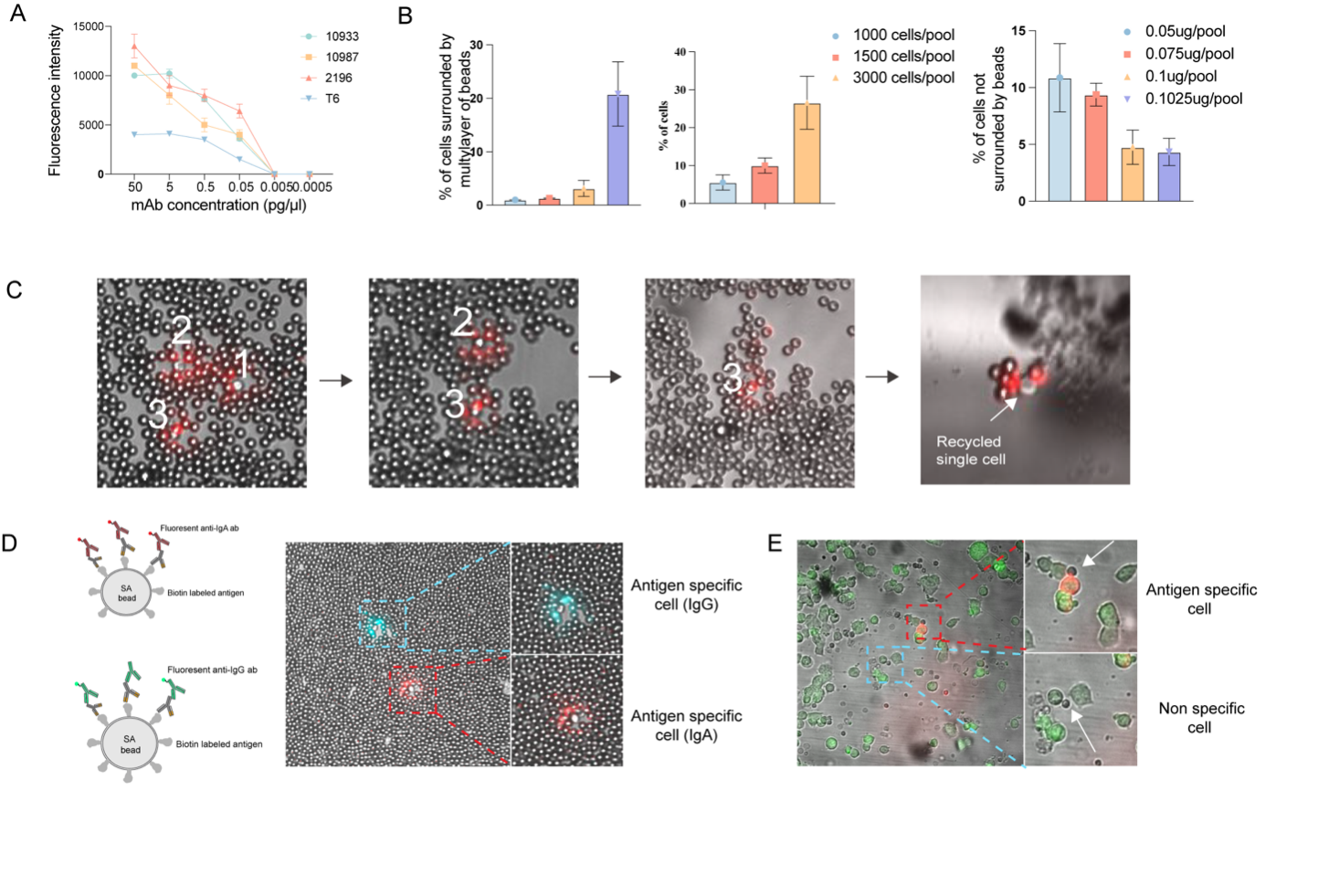


**Figure S2. Optimization of beads-based ASC screening. (A)** A curve graph depicting fluorescence intensity against antibody concentration. The average fluorescence intensity of five beads was selected for each condition, represented as mean ± SD. **(B)** Optimization of cell-bead distribution for single-cell assays. A total of 1500 cells/pool and 0.1 μg/pool was determined as the experimental condition for all subsequent experiments. **(C)** Presentation of the sequential recovery and release of three consecutive antigen-specific cells into microwells, demonstrating the stability of the system. **(D)** Identifying mAbs of different isotype. Left: Schematic illustrating detection of IgG and IgA isotypes on a streptavidin bead. Right: Color of halos indicating different isotype of antibodies secreted by ASC. **(E)** An example of a fluorescent halo formed by a B cell producing IgG specific for a receptor expressed on the surface of 293T cells.


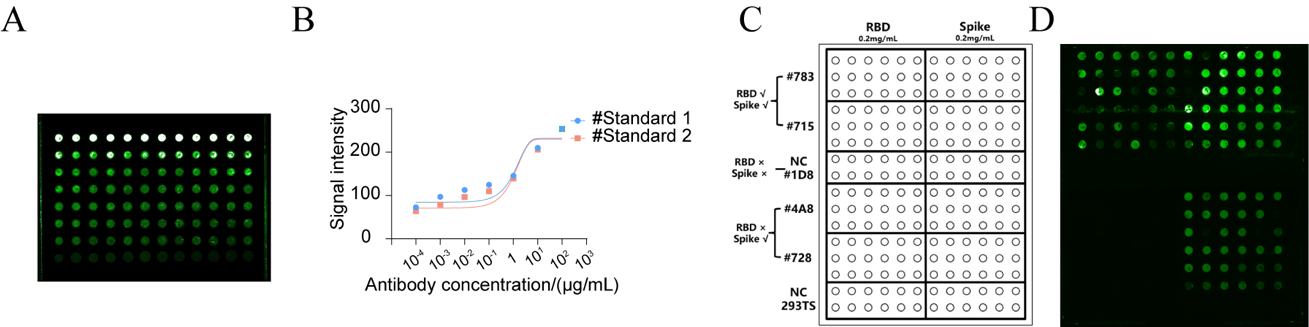


**Figure S3. Evaluation of on-glass ELISA. (A)** Scanning image of the glass slides for on-glass ELISA of each well for standards. **(B)** Comparison between standard curve obtained from plate assay and slide ELISA. Signal intensity was quantified using ImageJ and is represented as mean ± SD (n=12). **(C)** Partitioning slide regions for antigen incubation and corresponding antibody detection. **(D)** Scanning image from **(C)**


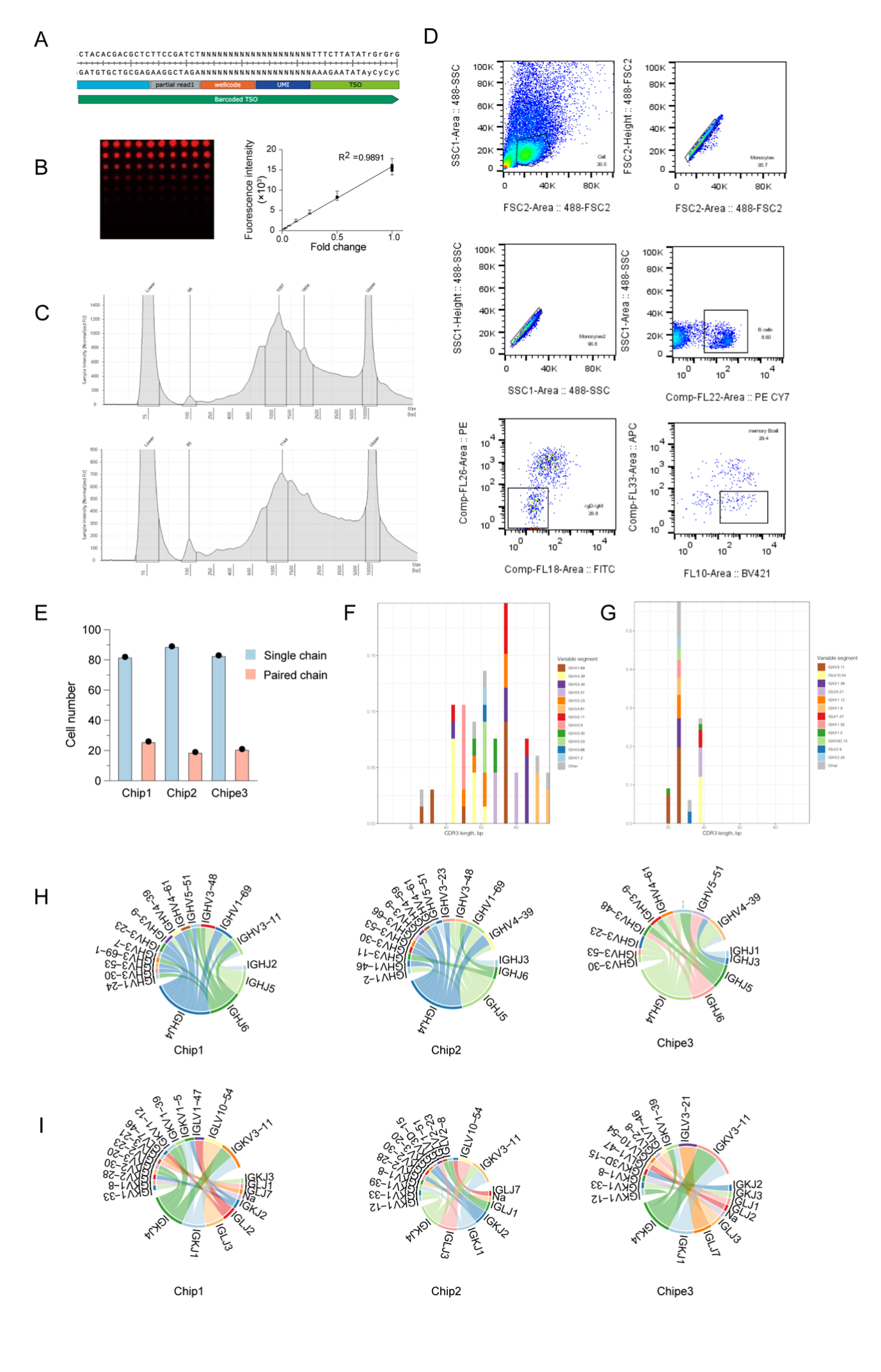


**Figure S4. Optimization of MoSMAR-seq.** **(A)** Structure and sequences used for barcoded TSO. **(B)** Assessment of fluorescent dye transferring via ultrasound. Fluorescence image (left) and the quantitative analysis curve(right) are shown after array scanning. Signal intensity was quantified using ImageJ and is represented as mean ± SD (n=10). **(C)** The distribution of pre-amplified cDNA fragments with (upper) or without (bottom) beads. **(D)** Gating strategy for isolating single memory B cells into MoSMAR-chip. **(E)** The number of cells with paired or single chains of BCR from three individual chips. **(F)** CDR3 length and gene usage distribution of heavy chains from sequenced memory B cells. **(G)** CDR3 length and gene usage distribution of light chains from sequenced memory B cells. **(H)** Chord diagrams illustrating V-J pairing of heavy chains from sequenced memory B cells. **(I)** Chord diagrams illustrating V-J pairing of light chains from sequenced memory B cells.


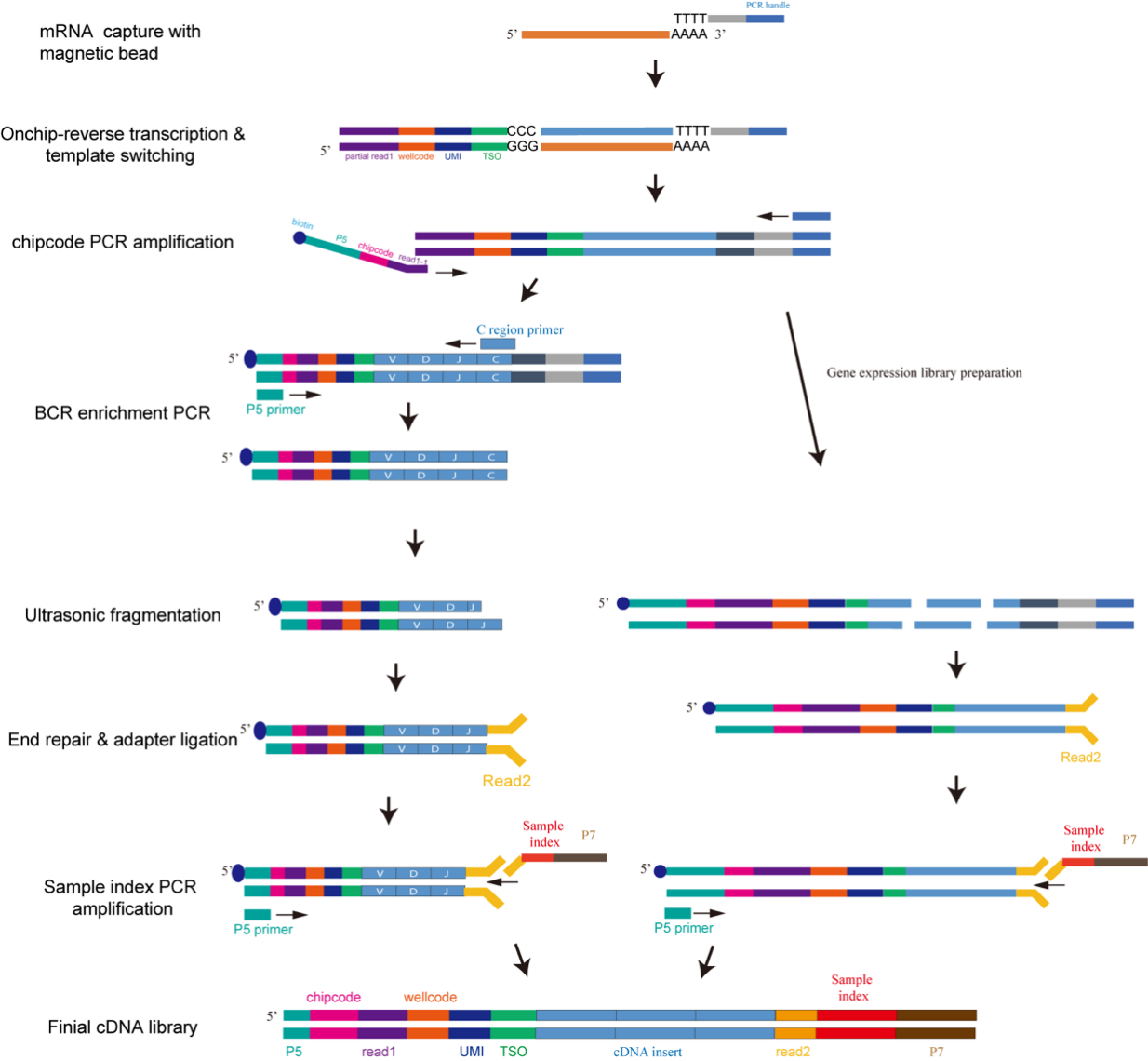


**Figure S5. Diagram illustrating the MoSMAR-seq protocol.** mRNA was first captured by the capture oligos in nanoliter droplets. Then, the mRNA was reverse transcribed to cDNA using the template switch method, followed by PCR amplification with P7 and TSO primers with chipcode. BCR sequences were amplificated with primers targeting conserved region of BCR gene and P5. The amplicons were purified, and then fragmented to ∼300 bp using ultrasound, followed by adapter ligation. Due to the biotinylated modification of the P5 primer, no adapter was added to the P5 ends. Finally, another round of amplification was performed to obtain the cDNA library for sequencing. The Read1 and Read2 primers were used to sequence the cDNA fragment and the wellcode/UMI, respectively.


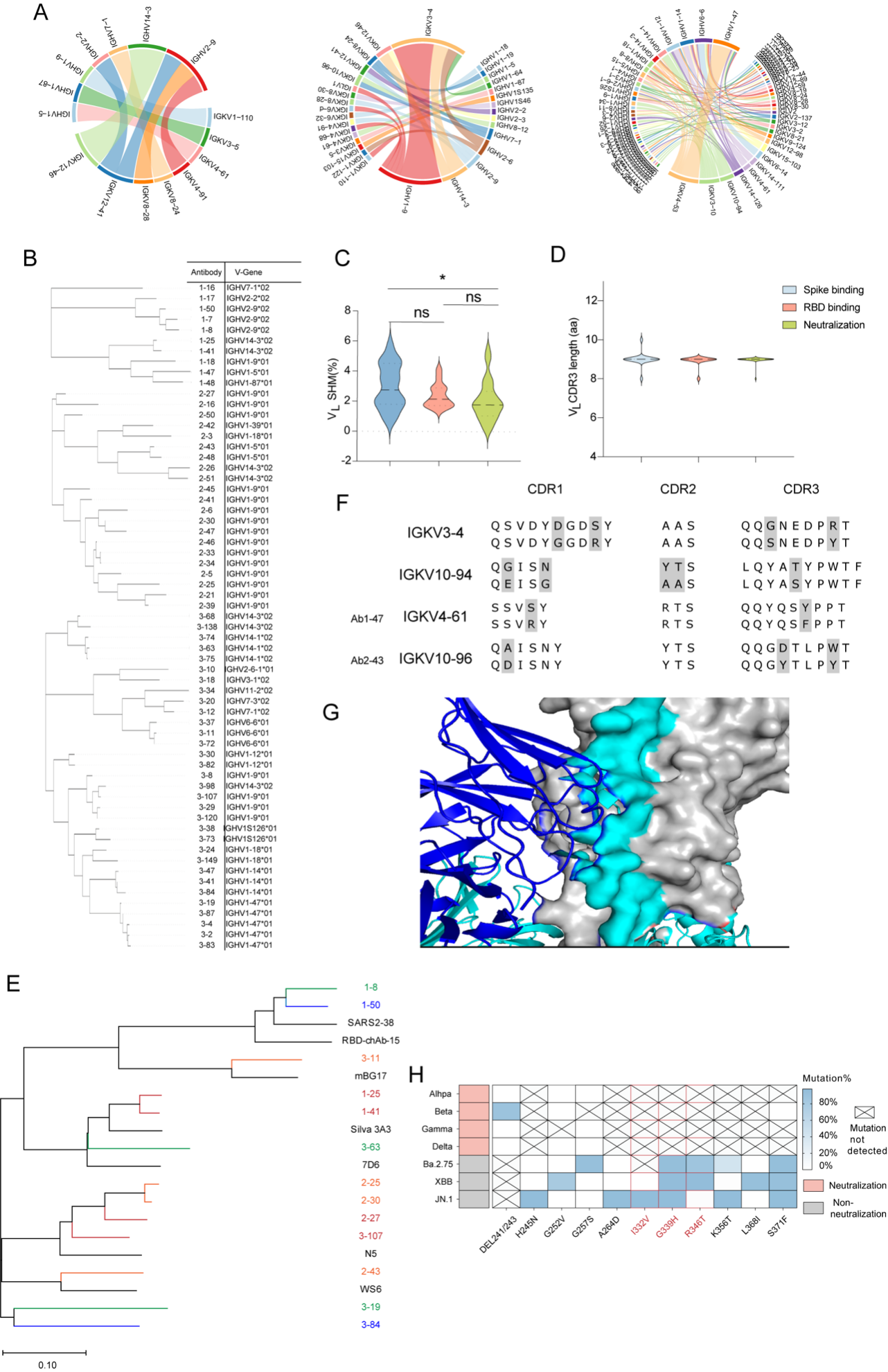


**Figure S6. Information of the gene sequence, characteristics, and structural prediction of sequenced LLPCs.** **(A)** Germline heavy and light gene usage and pairing among sequenced cells from mouse 1 (left), mouse 2 (middle), and mouse 3 (right) presented in chord diagrams. **(B)** Phylogenetic tree of MoSMAR-seq-identified sequences. **(C)** Violin plot showing somatic hypermutation of light chain from Spike binding, RBD binding, or neutralization groups. **(D)** Violin plot showing CDR3 length of light chain from Spike binding, RBD binding, or neutralization groups. **(E)** Phylogenetic tree of a public antibody cluster comprised of MoSMAR identiﬁed sequences and previously published SARS-CoV-2 antibody sequences from the CoV-AbDab database (black). **(F)** Amino acid sequence differences of light chain CDRs from public antibody clonotypes. **(G)** Predict antibody-antigen complex showing 2-43 binding with RBD region of spike protein. RBM of the RBD is colored with cyan, with otuer regions of RBD colored with grey. **(H)** Mutation prevalence of Spike protein of SARS-Cov-2 across lineages. Residue site of interacting with 2-27 was marked as red.


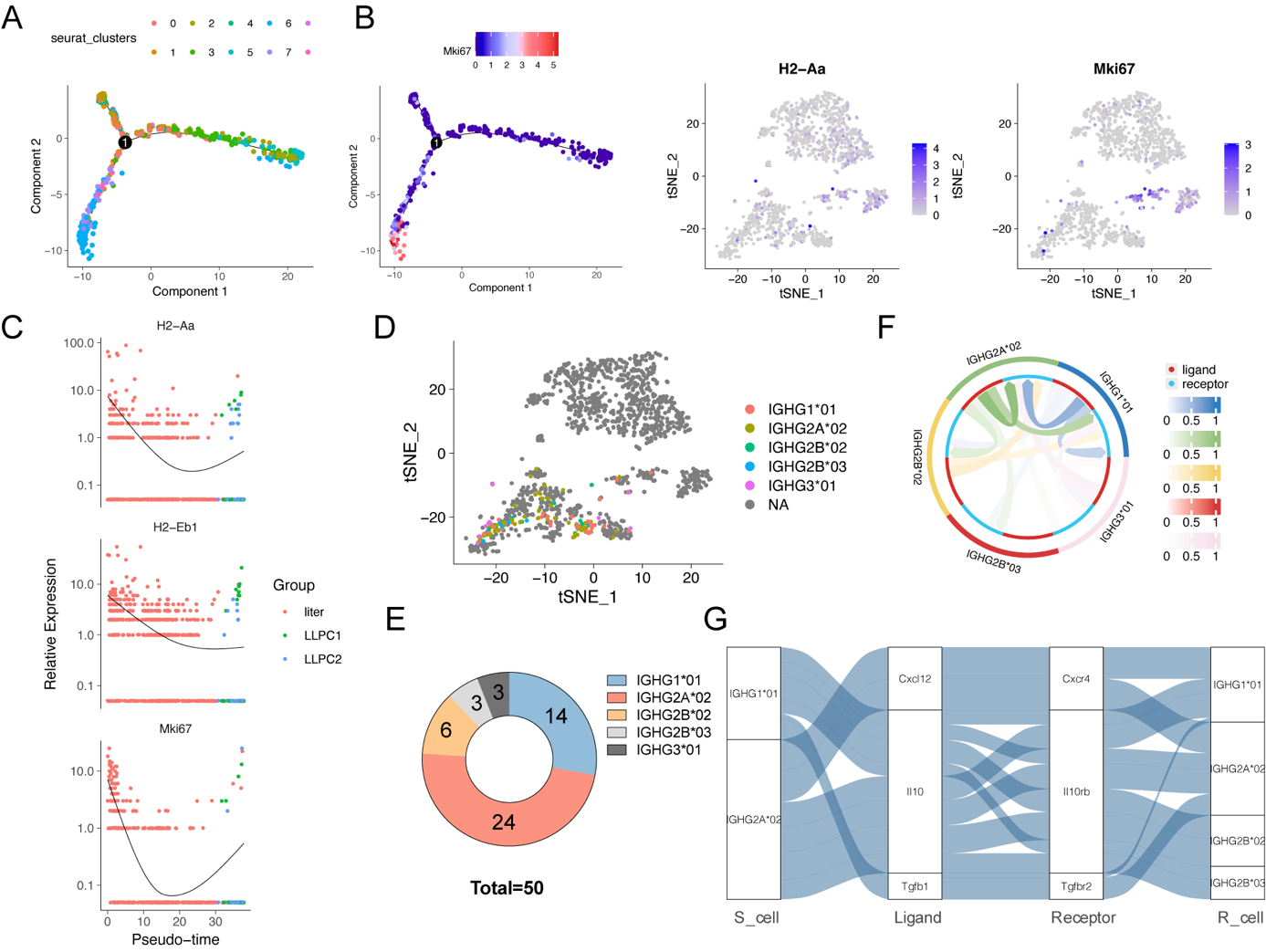


**Figure S7. Characterizing LLPCs with public dataset and ligand-receptor analyzing between LLPCs.**

**(A)** Pseudotime analysis of ASCs of different clusters. **(B) and (C)** Expression of Mki67 and major histocompatibility complex (MHC) class II genes in different BMPC clusters or sources. **(D)** Ig Subtype-Based t-SNE Cell Clustering Analysis. **(E)** Pie chart showing the proportion of antibody isotypes in antigen-specific B cells. **(F)** Interactive circus plots of different isotypes among sorted cells. **(G)** Sankey plot showing cellular interaction between IGHG1*01 and IGHG2A*02 cells.
